# The effects of oral probiotic intervention on brain structure and function in human adults: a systematic review

**DOI:** 10.1038/s41522-025-00872-x

**Published:** 2026-01-07

**Authors:** Ashley N. Hutchinson, Amanda E. Antonsson, Richard A. Forsgård, Julia König, John-Peter Ganda Mall, Julia Rode

**Affiliations:** 1 School of Medical Sciences, Faculty of Medicine and Health, Örebro University, Örebro, Sweden; 2 School of Health Sciences, Faculty of Medicine and Health, Örebro University, Örebro, Sweden

**Keywords:** Applied microbiology, Microbial communities

## Abstract

Oral intake of probiotics has been shown to positively impact depression, anxiety, stress and cognition. Recently, an effort was made to more objectively assess their impact on brain structure and function. However, there has been no exhaustive systematic assessment of outcomes of these studies, nor the techniques utilised. Therefore, we performed a systematic review on randomised, placebo-controlled trials assessing the effects of oral probiotic interventions on brain health by imaging or electrophysiology techniques in human adults. Of 2307 articles screened, 26 articles comprising 19 studies, totalling 762 healthy subjects or patients with various diseases, were ultimately included. The quality of most studies was high. Overall, probiotic intake appears to modify resting state connectivity and activity, decrease involvement of several brain regions during negative emotional stimulation, and improve sleep quality. Several studies found correlations between brain outcomes and clinical symptom ratings, supporting the relevance of brain imaging and electrophysiology techniques in this field.

## Introduction

Accumulating evidence suggests that the gut-brain axis, a complex, bidirectional communication system between the gastrointestinal tract and the central nervous system (CNS), influences human health. Much of the effect on gastrointestinal physiology can be attributed to the gut microbiota, an interconnected community of microorganisms that colonise the gut. The gut microbiota can mediate communication along the gut-brain axis via several key pathways including neural (such as stimulation of the vagus nerve^1^, the enteric nervous system (ENS)^2^, or spinal afferents^3^), humoral (such as the release of neurotransmitters^4^, tryptophan^5^, and short-chain fatty acids^6,7^), and immune pathways (via the release of cytokines and microbiota-immune system interactions^8^).

The microbiota and its metabolites have been implicated to play a role in brain development, function and health^9^. Preclinical studies in mice and rats have observed alterations in neurotransmitters^10,11^, brain structure^12^, and brain function^13–15^ in response to aberrations of the gut microbiota. The potential link between the gut microbiota, communication along the gut-brain axis, and brain health has been demonstrated in several human studies. For example, patients with irritable bowel syndrome (IBS) have been shown to have a similar microbiota composition as depressed patients, suggesting that alterations of the gut microbiota may be related to the pathogenesis of both disorders^16^. Furthermore, a prospective study found that individuals with higher anxiety and depression levels at baseline were more likely to develop IBS after one year compared to individuals with lower baseline levels^17^. Also, in individuals with IBS, cognitive behaviour therapy-related changes on resting state functional connectivity correlated with a gut microbiota change in a recent study^18^. Finally, the experts who determined the ROME IV diagnostic criteria for IBS have redefined IBS and other functional gastrointestinal disorders as “disorders of gut-brain interactions”, underscoring the relevance of this communication pathway^19^.

These findings suggest that modifying gut microbiota composition may be an attractive strategy to impact brain function and disease. One approach to such modification is through ingestion of prebiotics, compounds that can promote the growth of beneficial microbes by acting as an energy source, or probiotics, live microbial supplementation that confers a benefit to the host, when consumed in adequate amounts^20,21^. For example, a recent study investigated the effects of the prebiotic fibre inulin on brain activation in a reward-related, food decision-making task in overweight young adults^22^. In this two-week intervention, prebiotic ingestion resulted in decreased brain activation towards high-caloric food stimuli in the ventral tegmental area and the orbitofrontal cortex, as assessed by functional magnetic resonance imaging (fMRI). Focusing on probiotics, Groeger et al. found that an eight-week intervention with *Bifidobacterium longum* resulted in an improvement of psychometric measures in IBS patients with comorbid anxiety and/or depression^23^. In addition, several meta-analyses concluded that microbiota modifications by pre- and probiotics positively affect depressive symptoms, with the majority of studies focused on probiotics^24–26^, and psychiatric distress^24^.

While most evaluations of the effects of probiotics on brain health in humans are based on self-reported symptom ratings, the assessment of brain function via imaging techniques is a more objective method and advocated for by one of the aforementioned reviews^24^. Further systematic research using quantitative measurements to analyse potential changes in the human brain in response to probiotic interventions is needed. Examples of such quantitative measures to assess expected changes in both brain structure and function are techniques such as (f)MRI, diffusion tensor imaging (DTI), electroencephalography (EEG) and magnetoencephalography (MEG), amongst others. These non-invasive and in-depth measurements provide insights into morphometry, activity, and connectivity of the brain, offering a deeper understanding of potential changes due to modulation of the gut-brain axis. In addition, such insights contribute to the understanding of the interplay between complex human functions. Performing measurements during sleep^27^ or resting state can provide information about spontaneous fluctuations of the brain^28^, while performance of neurocognitive tasks can give insights about task-related brain activity or connectivity in order to observe stress or emotional responsiveness^29,30^.

As there is a growing number of studies assessing the effects of probiotics on brain outcomes, this systematic review assesses the effects of probiotic interventions in adults (of any study population, e.g. healthy or diseased) on brain function and structure assessed in randomised, placebo-controlled trials by brain imaging or electrophysiology technologies (including but not limited to (f)MRI, DTI, EEG, MEG).

## Results

### Trial selection

The literature search (with the latest update in August 2025) resulted in a total of 2943 records, of which 2307 records were screened, resulting in 45 articles that were included in a full-text assessment for eligibility. Of these, 26 articles originating from 19 studies were ultimately included in this review (Table 1, Supplementary Fig. 2).

**Table 1:** Overview of included studies

Primary publication; last name first author, publication year/additional publications; last name first author, publication year/country	Study design*/length of intervention/recruitment years	Population description	Total number of participants/number of participants in neuroimaging analysis (Probiotic/Placebo)	Neuroimaging techniques used	Neuroimaging outcomes reported	Neuroimaging aim/hypothesis of the study	Primary aim/hypothesis****
Malaguarnera et al. 2007^49^ Italy	Parallel arm 90 days Not stated	Cirrhotic patients, diagnosed with hepatitis B, hepatitis C, or cryptogenic cirrhosis, mean age 45.5 years**:	60/60 (30/30)	EEG	Brain activity	To evaluate the effects of the probiotic intervention with the addition of fructo-oligosaccharides on cognitive function.	To assess the clinical efficacy of the probiotic plus fructo-oligosaccharides in the treatment of minimal hepatic encephalopathy.
Tillisch et al. 2013^31^ USA	Parallel arm 4 weeks 2009–2010	Healthy adult women, 18–55 years.	36/23 (12/11)***	fMRI	Resting state functional connectivity, Task-related functional connectivity	To investigate if the probiotic intervention altered brain intrinsic connectivity or responses to emotional attention tasks in healthy women.	Same as the aim of neuroimaging.
Kelly et al. 2017^34^ Ireland	Crossover 4 weeks Not stated	Healthy male participants, 18–40 years.	29/29 (29/29)**	EEG	Brain activity	To investigate the effect of the probiotic intervention on brain activity patterns in frontal, parietal and central regions.	To determine the impact of the probiotic intervention on stress-related behaviours, physiology, inflammatory response, cognitive performance and brain activity patterns in healthy male participants.
Pinto-Sanchez et al. 2017^50^ Martin et al. 2024^51^ Canada	Parallel arm 6 weeks 2011–2014	Adult patients with Irritable Bowel Syndrome (Rome III criteria) and mild to moderate anxiety and/or depression ([HAD-anxiety] or [HAD-depression] score 8–14), 21–65 years.	44/38 (18/20)	fMRI	Task-related brain activity	To explore the effect of the probiotic intervention on brain activation patterns.	To evaluate the effects of the probiotic intervention on anxiety and depression in patients with IBS.
Takada et al. 2017^36^ Japan	Parallel arm 11 weeks Not stated	Healthy medical students, preparing for qualification examinations, under 30 years.	98/94 (48/46)	EEG	Sleep EEG	To examine whether the probiotic intervention improves sleep quality under psychological stress.	Same as the aim of neuroimaging.
Bagga et al. 2018^37^ Bagga et al. 2019^38^ Austria	Parallel arm 4 weeks Not stated	Healthy adults, 20–40 years.	45/30 (15/15)***	MRI (DTI); fMRI	Structural connectivity; Resting state functional connectivity, Task-related brain activity	Bagga 2018: To investigate the effects of the probiotic intervention on brain function in healthy volunteers. Bagga 2019: To investigate the effects of the probiotic intervention on whole-brain functional and structural connectivity in healthy volunteers.	To investigate the effects of the probiotic intervention on behaviour, brain function and gut microbial composition in healthy volunteers.
Nakagawa et al. 2018^45^ Japan	Crossover 4 weeks Not stated	Adults who have a problem with everyday sleep, 20–64 years.	40/21 (unclear how many from each group)	EEG	Sleep EEG	To examine the effects of the probiotic intervention on sleep EEG, as part of assessing the sleep quality.	To investigate the effect of the probiotic intervention on the quality of sleep.
Papalini et al. 2019^32^ Bloemendaal et al. 2021^33^ the Netherlands	Parallel arm 28 days Not stated	Healthy adult women using hormonal contraceptives, 18–40 years.	61/58 (29/29)	fMRI	Task-related brain activity	Papalini 2019: To investigate the effects of the probiotic intervention on specific neurocognitive measures of emotion reactivity, emotion regulation, and cognitive control. In addition, testing whether probiotics can buffer against the detrimental effects of acute stress on working memory. Bloemendaal 2021: To assess gut microbial composition alterations and their association with stress-induced working memory changes after the probiotic intervention.	To investigate the effects of the probiotic intervention on specific neurocognitive measures of emotion reactivity, emotion regulation, and cognitive control using fMRI. In addition, to assess whether probiotics can buffer against the detrimental effects of acute stress on working memory.
Wang et al. 2019^39^ Germany	Parallel arm 4 weeks 2016–2018	Healthy adults, 18–50 years.	43/40 (20/20)	MEG	Resting state brain activity, Task-related brain activity	To examine the effects of the probiotic intervention in neural responses to social stress.	Same as the aim of neuroimaging.
Adikari et al. 2020^35^ Malaysia	Parallel arm 8 weeks Not stated	Young adult male football players, 18–21 years.	19/19 (10/9)	EEG	Brain activity	To investigate the effects of the probiotic intervention on the electrical activities of the brain during a cognitive task among competitive football players.	To determine the effect of the probiotic intervention on anxiety-induced physiological parameters among competitive football players.
Ho et al. 2021^46^ Taiwan	Parallel arm 30 days 2018–2019	Adults with self-reported insomnia, 20–40 years.	40/40 (21/19)	EEG	Brain activity	To determine whether the probiotic intervention improves sleep quality.	To determine whether the probiotic intervention reduces the severity of anxiety and depressive symptoms, regulates autonomic nervous system function, and improves sleep quality.
Asaoka et al. 2022^48^ Japan	Parallel arm 24 weeks 2018–2020	Elderly with suspected mild cognitive impairment, 65–89 years.	130/89 (42/47)	MRI	Morphometry	To investigate the effect of the probiotic intervention in enhancing cognition and preventing brain atrophy of older patients with mild cognitive impairment.	Same as the aim of neuroimaging.
Ascone et al. 2022^40,81^ Germany	Parallel arm 28 days 2018–2019	Healthy adults, 18–40 years.	67/59 (30/29)	MRI; fMRI	Morphometry; Resting state functional connectivity	To test the hypothesis that the probiotic intervention is associated with enhanced hippocampal regional grey matter volume, as well as hippocampal functional connectivity.	Same as neuroimaging aim, and related improvements in amnestic and navigational performance were expected to be observed in healthy human volunteers.
Edebol-Carlman et al. 2022^41^ Rode et al. 2022^42^ Rode et al. 2022^43^ Sweden	Crossover 4 weeks 2018	Healthy adults, 18–65 years.	22/22 (22/22)**	MRI; fMRI	Morphometry; Resting state functional connectivity, Task-related functional connectivity, Task-related brain activity	Edebol Carlman 2022: To investigate if the probiotic intervention affects stress processing. Rode 2022: To investigate if the probiotic intervention affects functional brain responses in healthy subjects during an emotional attention task. Rode 2022: To investigate the neural effect of the probiotic intervention on resting state functional connectivity and voxel-based morphometry.	Same as neuroimaging aim in Rode 2022.
Schaub et al. 2022^53^ Yamanbaeva et al. 2023^54^ Schneider et al. 2023^55^ Switzerland	Parallel arm 31 days 2017–2020	Adult patients within inpatient care receiving treatment as usual for depression, over 18 years. No upper age limit stated.	60 (whereof 47 completed intervention)/32 (14/18)	MRI (incl. DTI, ASL); fMRI	Morphometry; Structural connectivity; Cerebral blood perfusion; Resting state functional connectivity, Task-related brain activity	Schaub 2022: To investigate the effects of the probiotic add-on therapy on neural changes. Yamanbaeva 2023: Investigate the effects of the probiotic add-on therapy on fronto-limbic brain structure, function, and perfusion in major depressive disorder and whether these effects were related to symptom changes. Schneider 2023: To investigate the effects of the probiotic add-on therapy on cognition and related brain functions in major depressive disorder.	To determine whether short-term, high-dose probiotic intervention reduces depressive symptoms along with gut microbial and neural changes in depressed patients.
Kikuchi-Hayakawa et al. 2023^47^ Japan	Crossover 4 weeks 2021	Office workers who were aware of impaired sleep quality, 40–59 years.	12/12 (12/12)	EEG	Brain activity	To explore the effect of the probiotic intervention on physiological state indicators in healthy office workers with sleep complaints via EEG.	To explore the possible efficacy of the probiotic intervention for improving daytime performance in healthy participants with sleep complaints. Assessing subjective mood using perceived mood questionnaires and measuring physiological indicators via EEG and heart rate variability.
Mutoh et al. 2024^44^ Japan	Parallel arm 6 weeks 2021	Healthy adults, 20–64 years.	30/30 (15/15)	fNIRS	Brain activity, Laterality	To evaluate the effects of the probiotic intervention on mood states in humans via NIRS assessment.	To investigate the potential of the probiotic intervention to improve mood in humans via NIRS assessment, questionnaires, and measurements of gut microbiota and metabolites.
Ranisavljev et al. 2024 & 2025^52,57^ Serbia	Parallel arm 3 months 2022–2023	Adults with post-COVID-19 ME/CFS, 18–65 years.	26/11 (6/5)	Proton MRS	Brain tissue levels of choline, creatine, and N-acetyl aspartate	To evaluate the effects of the synbiotic intervention on tissue metabolism in patients with post-COVID-19 ME/CFS.	The primary endpoint was the change in general fatigue from baseline to the 3-month follow-up.
Li et al. 2025^56^ China	Parallel arm 30 days 2023–2024	Adult clinical patients with MDD with anhedonic symptoms, 18–65 years.	71/55 (27/28)	EEG	Brain activity	To investigate the effects of the probiotic intervention on the neural activities associated with reward expectation and consumption.	To assess the efficacy of the probiotic intervention in alleviating anhedonia in patients with MDD, utilising both objective and subjective assessments.

*ASL* arterial spin labelling MRI; *DTI* diffusion tensor imaging; *EEG* electroencephalography; *(f)MRI* (Functional) magnetic resonance imaging; *(f)NIRS* (Functional) near-infrared spectroscopy; *MEG* magnetoencephalography; *MRS* magnetic resonance spectroscopy; *IBS* irritable bowel syndrome; *post-COVID-19 ME/CFS* post-COVID-19 myalgic encephalomyelitis/ chronic fatigue syndrome; *MDD* major depressive disorder. *All studies are randomised and placebo-controlled; **Actual mean age of study population, since the inclusion criteria were not provided; ***Study also included a no intervention group, which is not reported in this review; ****Primary aim as stated in the abstract, in case of several publications from one study, the abstract of the earliest published article was used for reference if authors did not state more clearly which of their publications consulted the primary aim or did not state the primary aim more clearly in introduction or methods section.

### Study participants

The 19 studies included a total of 762 healthy subjects or patients with various diseases. Ten of the studies included healthy individuals, of which two studies included women only^31–33^, two studies included men only^34,35^, and six studies included both women and men^36–44^. Of the studies conducted in healthy individuals, Adikari et al. only included young male football players (18–21 years of age)^35^, while Takada et al. specifically included medical students preparing for a national qualification examination^36^.

The remaining studies focused on specific health conditions or diagnoses. Three studies included individuals with sleep problems^45–47^. The other studies included elderly participants with suspected mild cognitive impairment^48^, cirrhotic patients^49^, patients with IBS with comorbid anxiety or depression^50,51^, patients with post-COVID-19 myalgic encephalomyelitis/chronic fatigue syndrome (ME/CFS)^52^ and patients with depression undergoing an active anti-depressant treatment plan^53–56^.

### Probiotic interventions

Of the 19 studies included in this review, 12 studies investigated a product with a single probiotic strain^34–36,39,44–51,56^, whereas seven studies employed a multi-strain product^31–33,37,38,40–43,52–55^ (Table 2). Collectively, these studies included 37 individual strains from 15 different species of 9 bacterial genera. Products containing *Bifidobacterium* species were investigated in 12/19 studies, *Lacticaseibacillus* (9/19), *Lactiplantibacillus* (6/19), *Lactobacillus* (7/19), *Levilactobacillus* (1/19), *Ligilactobacillus* (2/19), *Lactococcus* (3/19), *Streptococcus* (3/19) and *Pediococcus* (1/19). On the species level, the most commonly utilised species were *L. paracasei* (7/19), *L. plantarum* (6/19), *B. lactis* (6/19), *B. longum* (5/19), *L. acidophilus* (4/19), and *L. helveticus* (4/19). The daily bacterial doses ranged from 7.0 × 10^7^ to 1.0 × 10^11^ colony-forming units (CFU); however, in some cases, the exact doses of individual bacterial strains in the multi-strain product were not reported^31–33,37,38,40,53–55^. In addition, one study^45^ reported the daily dose as grams of the product, not as CFU, leaving the number of bacteria in the product uncertain.

**Table 2:** Probiotic interventions

Study	Study design	Probiotic strain(s)	Daily dose	Intervention period	Medium	Placebo treatment	Compliance
Studies analysing single-strain products							
Malaguarnera et al.^49^	Parallel arm	*Bifidobacterium longum* W11	5.0 × 10^9^ CFU	90 days	Powder containing 2.5 g fructo-oligosaccharides and vitamins B1, B2, B6 and B12 dissolved in water or milk	Vitamins B1, B2, B6 and B12 dissolved in water or milk^#^	Compliance cutoff 80–120%
Kelly et al.^34^	Cross-over	*Lacticaseibacillus rhamnosus*	1.0 × 10^9^ CFU	4 weeks	Capsule containing corn starch, magnesium stearate, and silicon dioxide	Capsule containing corn starch, magnesium stearate, and silicon dioxide without the probiotic strain*	Not reported
Pinto-Sanchez et al.^50^ Martin et al.^51^	Parallel arm	*Bifidobacterium longum* NCC3001	1.0 × 10^10^ CFU	6 weeks	Maltodextrin-based powder dissolved in lactose-free milk	Maltodextrin-based powder without the probiotic strain dissolved in lactose-free milk*	Checked but not reported
Takada et al.^36^	Parallel arm	*Lacticaseibacillus casei* Shirota YIT 9029	1.0 × 10^11^ CFU	11 weeks	Fermented milk	Milk with added lactic acid without the probiotic strain^#^	Placebo: 97.0% Probiotic: 97.5%
Nakagawa et al.^45^	Cross-over	*Lactobacillus helveticus* MIKI-020	200 mg of live lactic acid bacteria powder, 8 times/day	4 weeks	Tablets containing 900 mg of fermented products of *L. helveticus* MIKI-020, 200 mg of ground lactic acid bacteria powder, and 200 mg of theanine (and maltitol, lactose, calcium stearate, lactic acid, flavour, yeast extract)	Tablets without the probiotic strain, probiotic-derived products, and theanine (and maltitol, lactose, calcium stearate, lactic acid, flavor, yeast extract)^#^	Compliance <75% led to exclusion from the study
Wang et al.^39^	Parallel arm	*Bifidobacterium longum* 1714^TM^	1.0 × 10^9^ CFU	4 weeks	Maltodextrin-based powder dissolved in 50 mL of water	Maltodextrin-based powder without the probiotic strain dissolved in 50 mL of water*	Not reported
Adikari et al.^35^	Parallel arm	*Lacticaseibacillus casei* Shirota	3.0 × 10^10^ CFU	8 weeks	Cultured milk (80 mL) mixed with orange fruit juice (120 mL)	200 mL of orange fruit juice^#^	Not reported
Ho et al.^46^	Parallel arm	*Lactiplantibacillus plantarum* PS128	3.0 × 10^10^ CFU	30 days	Microcrystalline cellulose capsules	Microcrystalline cellulose capsules without the probiotic strain*	Compliance >98%
Asaoka et al.^48^	Parallel arm	*Bifidobacterium breve* MCC1274	2.0 × 10^10^ CFU	24 weeks	Mainly maize starch-based powder	Maize starch-based powder without the probiotic strain^#^	Placebo: 96.1 ± 5.2% Probiotic: 97.2 ± 3.5%
Kikuchi-Hayakawa et al.^47^	Cross-over	*Lacticaseibacillus paracasei* Shirota YIT 9029	1.0 × 10^11^ CFU	4 weeks	Fermented milk	Non-fermented milk with added lactic acid without the probiotic strain^#^	Placebo: 99.7% Probiotic: 100%
Mutoh et al.^44^	Parallel arm	*Bifidobacterium breve* M-16V	2.0 × 10^10^ CFU	6 weeks	Maltodextrin-based powder formed into a stick	Maltodextrin-based powder^+^	Placebo: 99.1% Probiotic: 97.9%
Li et al.^56^	Parallel arm	*Pediococcus acidilactici* CCFM6432	1.0 × 10^9^ CFU	30 days	Powder, consumed directly or dissolved in water	Powder^+^	Checked but exact details not reported (6 participants were excluded due to compliance issues)
Studies analysing multi-strain products							
Tillisch et al.^31^	Parallel arm	*Bifidobacterium lactis*	1.25 × 10^10^ CFU, 2 doses/day	4 weeks	Fermented milk	A non-fermented milk without probiotics^+^	Compliance <75% led to exclusion from the study
		*Lactobacillus bulgaricus*	1.2 × 10^9^ CFU, 2 doses/day				
		*Lactococcus lactis*	Not reported, 2 doses/day				
		*Streptococcus thermophilus*	1.2 × 10^9^ CFU, 2 doses/day				
Bagga et al.^37^ Bagga et al.^38^	Parallel arm	*Bifidobacterium bifidum* W23	7.5 × 10^9^ CFU	4 weeks	Freeze-dried powder of maize starch and maltodextrin dissolved in milk or lukewarm water	Freeze-dried powder of maize starch and maltodextrin without the probiotic strains dissolved in milk or lukewarm water^+^	Checked but not reported
		*Bifidobacterium lactis* W51					
		*Bifidobacterium lactis* W52					
		*Lacticaseibacillus casei* W56					
		*Lacticaseibacillus paracasei* W20					
		*Lactiplantibacillus plantarum* W62					
		*Lactobacillus acidophilus* W22					
		*Lactococcus lactis* W19					
		*Ligilactobacillus salivarius* W24					
Papalini et al.^32^ Bloemendaal et al.^33^	Parallel arm	*Bifidobacterium bifidum* W23	5.0 × 10^9^ CFU	28 days	Powder consisting of maize starch, maltodextrin, vegetable protein and a mineral mix dissolved in water or milk	Powder consisting of maize starch, maltodextrin, vegetable protein and a mineral mix dissolved in water or milk*	Not reported
		*Bifidobacterium lactis* W51					
		*Bifidobacterium lactis* W52					
		*Lacticaseibacillus paracasei* W56					
		*Lactobacillus acidophilus* W37					
		*Lactococcus lactis* W19					
		*Lactococcus lactis* W58					
		*Levilactobacillus brevis* W63					
		*Ligilactobacillus salivarius* W24					
Ascone et al.^40,81^	Parallel arm	*Bifidobacterium breve*	4.5 × 10^9^ CFU	28 days	Maltose-based powder dissolved in water, milk, juice or similar	A commercially available baby milk-powder dissolved in water, milk, juice or similar^#^	Not reported
		*Bifidobacterium lactis* NCIMB 30435					
		*Bifidobacterium lactis* NCIMB 30436					
		*Lacticaseibacillus paracasei*					
		*Lactiplantibacillus plantarum*					
		*Lactobacillus acidophilus*					
		*Lactobacillus helveticus*					
		*Streptococcus thermophilus*					
Edebol Carlman et al.^41^ Rode et al.^42^ Rode et al.^43^	Cross-over	*Bifidobacterium longum* R0175	7.0 × 10^7^ CFU	4 weeks	Inulin- and fructose-based powder with zinc, magnesium, potassium, glutathione, lactoferrin, sweeteners, dyes and aroma dissolved in a glass of water	Fructose-based powder with sweeteners, dyes and aroma dissolved in a glass of water^#^	Compliance >90%
		*Lactiplantibacillus plantarum* R1012	8.0 × 10^8^ CFU				
		*Lactobacillus helveticus* R0052	2.0 × 10^9^ CFU				
Schaub et al.^53^ Yamanbaeva et al.^54^ Schneider et al.^55^	Parallel arm	*Bifidobacterium breve* NCIMB 30441	9.0 × 10^9^ CFU	31 days	Maltose-based powder dissolved in any cold, non-carbonated drink	Maltose-based powder without the probiotic strains dissolved in any cold, non-carbonated drink*	Placebo: 86% Probiotic: 83%
		*Bifidobacterium lactis* NCIMB 30435					
		*Bifidobacterium lactis* NCIMB 30436					
		*Lacticaseibacillus paracasei* NCIMB 30439					
		*Lactiplantibacillus plantarum* NCIMB 30437					
		*Lactobacillus acidophilus* NCIMB 30442					
		*Lactobacillus helveticus*					
		*Streptococcus thermophilus* NCIMB 30438					
Ranisavljev et al.^52,57^	Parallel arm	*Lacticaseibacillus rhamnosus* DSM 32550	5.1 × 10^9^ CFU at study start, 1.4 × 10^9^ CFU at study end; input ratio of the different strains was 13.3: 3.6: 8.0: 1	3 months	Powder containing 2.5 g fructo-oligosaccharides, 5 mg zinc, maltodextrin dissolved in lukewarm water	Maltodextrin dissolved in lukewarm water^#^	Not reported
		*Lactiplantibacillus plantarum* DSM 34532					
		*Bifidobacterium lactis* DSM 32269					
		*Bifidobacterium longum* DSM 32946					

*CFU* Colony-forming units. This table is sorted by publication year and alphabetical order of first author; for studies comprised of several articles, the first published was considered as the reference point. The names of some probiotic strains were updated according to the recently adopted nomenclature and taxonomy^82^. Additional information, e.g., on commercial product name, product manufacturer and study funding source, can be found in Supplementary Table 8. *identical composition without probiotic(s), ^#^clearly different composition, not only without probiotic(s), ^+^comparison of products not entirely clear by information provided.

In addition to the wide variety of strains utilised, the intervention length, mode of delivery, placebo utilised, and compliance reporting strategy varied among the included studies. The length of the intervention periods ranged from 28 to 90 days, with most studies (12/19) lasting for approximately four weeks. The products were consumed as a powder with a carbohydrate-based carrier (11/19), as a fermented milk drink (4/19), or as capsules (3/19). Only 6/19 studies reported truly identical placebo products, containing the exact same ingredients except for the addition of the probiotic strains, 9/19 were overtly not comparable, and for 4/19 the quality of the comparison could not be determined from the information provided. Notably, in two studies, the probiotic product included 2.5 g fructo-oligosaccharide, a known prebiotic, which was not present in the placebo treatment^49,52,57^. Furthermore, in another study, the probiotic tablets but not the placebo contained theanine, an amino acid that is known to affect the CNS^45^. When considering compliance, 6/19 studies reported the exact proportions of compliant participants^36,41–44,46,47,53–55^. In these studies, compliance was high (83–100%). A total of 4/19 studies reported cut-off compliance values for inclusion in the final data analysis^31,45,49,53–55^, and 9/19 studies did not report any values on compliance^32–35,37–40,48,50–52,56^.

Additional information on probiotic interventions, such as commercial product name, product manufacturer and study funding source, can be found in Supplementary Table 8.

### Structural brain outcomes

Structural connectivity. Structural connectivity, i.e., anatomical connections mostly by bundles of neuronal axons, was assessed in two of the included studies using diffusion tensor imaging (DTI). While Bagga et al. found no effects of a multi-strain probiotic intervention on fractional anisotropy and mean diffusivity (two measures of DTI) in a study population of healthy young adults^38^, Yamanbaeva et al. reported increased post-intervention mean diffusivity in the right uncinate fasciculus (a white matter fibre tract) after placebo but not probiotic intervention^54^. They also observed typical negative correlations between fractional anisotropy and mean diffusivity solely in the probiotics group^54^.

Morphometry. Morphometric effects were assessed in four of the included studies using voxel-based or surface-based morphometry techniques^40,43,48,53^. On the whole-brain level, Schaub et al. reported increased grey matter volume in the calcarine and lingual sulcus, both associated with the visual cortex (without differences in thickness, gyrification or sulcus depth) after any intervention, but no intervention-related differences^53^. Furthermore, Rode et al. observed lower grey matter volume in a cluster covering the left supramarginal gyrus and superior parietal lobule (both associated with emotion, attention, and memory) after probiotic compared to placebo intervention^43^. White matter volume seems to have been not affected, as reported in the methods description, but not described in the results section^43^. However, no intervention group*time effects were observed for grey matter volume in the studies by Schaub et al.^53^ and Ascone et al.^40^, nor for white matter volume in the study by Ascone et al.^40^. Moreover, Ascone et al. did not find intervention group*time effects on the hippocampal structure^40^. In addition to investigating changes in grey and white matter volume, Asaoka et al. specifically investigated brain atrophy changes using a modified voxel-based morphometry technique and detected an increase in grey matter atrophy in the whole brain (which also differed significantly between the intervention groups) and in the volume-of-interest for Alzheimer’s disease upon placebo but not probiotic intervention^48^. The differences were dependent on the brain atrophy severity at baseline^48^.

### Functional brain outcomes

Resting state functional magnetic resonance imaging (rsfMRI). Seven studies assessed brain function using fMRI tools. Out of those, four studies (all utilising four-week multi-strain probiotic interventions) employed rsfMRI^38,40,43,54^ (Table 3). A fifth study performing rsfMRI did not report on the obtained results separately but instead used it for correlation with task-based fMRI^31^ (Table 3). The scanning duration varied from five to ten minutes, and participants were instructed to have their eyes open^40^, closed^31,43^, or it was not specified^38,54^. The data analyses were conducted with targeted or untargeted approaches.

**Table 3:** Probiotic effects on resting state functional connectivity

Study	Data analyses approach	Study design	Direction of effect	Localisation of effect
Bagga et al.^38^	ICA, GLM, whole-brain	Parallel groups, baseline and 4 weeks	Probiotic < placebo	default mode network (frontal pole, superior frontal gyrus and paracingulate gyrus) and in pre- and postcentral gyri.
Rode et al.^43^	SBC with several networks as seeds (default mode, salience, frontoparietal, language, cerebellar, dorsal attention, sensorimotor, visual)	Cross-over, 4 weeks, (no baseline)	Probiotic < placebo	Left supramarginal gyrus to left postcentral gyrus;
			Probiotic < placebo	Right supramarginal gyrus to precuneus cortex, bilateral intracalcarine cortex, bilateral supracalcarine cortex; Right cerebellum
			Probiotic < placebo	Left posterior parietal cortex to the left middle frontal gyrus, left precentral gyrus
			Probiotic > placebo	Medial prefrontal cortex to right postcentral gyrus, right superior parietal lobule
			Probiotic > placebo	Right inferior frontal gyrus to middle temporal gyrus (temporooccipital part right), inferior temporal gyrus (temporooccipital part right), lateral occipital cortex (inferior division right)
Ascone et al.^40^	SBC, flexible factorial design, seeds: bilateral, right, left hippocampus	Parallel groups, baseline, 4 weeks	No significant main effects, no time*group interactions	-
Yamanbaeva et al.^54^	SBC, 2×2 mixed ANOVA, combined seed of: subcallosal cortex/subgenual anterior cingulate cortex, hippocampus, amygdala, temporal pole, insula, orbitofrontal cortex. The resulting two clusters in the precuneus and the left superior parietal lobule/supramarginal gyrus were used for post hoc tests.	Parallel groups, baseline, 31 days significant time*group interactions	Probiotic < placebo	left superior parietal lobule/supramarginal gyrus to subcallosal cortex, right amygdala, left hippocampus
			Probiotic > placebo	left superior parietal lobule/supramarginal gyrus to left orbitofrontal cortex
			Probiotic > placebo	precuneus to subcallosal cortex*, right* & left amygdala, bilateral hippocampus, left temporal pole (*not significant after correction for baseline differences)
Tillisch et al.^31^ **	MVA, seeds periaqueductal grey, somatosensory cortex, insula) derived from emotional task-based activity	Parallel groups, baseline, 4 weeks	Correlations with task-induced regional brain activity and connectivity	Periaqueductal grey (part of midbrain region) to thalamus, insula, cingulate gyrus, amygdala, hippocampus, parahippocampal gyrus, basal ganglia, region BA40

*ANOVA* analysis of variance, *GLM* generalised linear model, *ICA* independent component, *MVA* multivariate analysis, *SBC* seed based connectivity analysis. **described in section *Correlation between fMRI outcomes*

In a healthy population, Bagga et al. observed significantly lower resting functional connectivity, i.e., synchronised brain activity, in the probiotics compared to the placebo group in the default mode network — the typical resting state brain network — and in pre- and postcentral gyri^38^. In a similar healthy population, Rode et al. reported altered (increased and decreased) resting state functional connectivity after probiotics compared to placebo intervention between regions of the default mode network and regions involved mainly in attention and motor functions, amongst others^43^. Ascone et al. did not find any probiotic effect on resting state functional connectivity originating from bilateral hippocampal regions (mainly associated with memory functions) to any other part of the brain^40^.

In a clinically depressed patient cohort, Yamanbaeva et al. reported significant time*group interactions deriving from increased resting state functional connectivity after probiotics intervention and decreased connectivity post-placebo from the precuneus (a brain region with a broad spectrum of highly-integrated tasks)^54^. Furthermore, they also observed altered connectivity post-probiotics from the left superior parietal lobule/supramarginal gyrus (regions involved in emotion, attention and memory)^54^. Most of those functional connectivity alterations remained significant even after correcting for significant baseline differences^54^.

Task-based fMRI. The tasks performed during fMRI acquisition were of emotional and/or cognitive nature with a possible stress component in all six studies^31,32,37,41,42,50,53,55^ (Table 4). Despite some overlap in study and emotional paradigm design, the results were heterogenous with little consistency across the different studies. Several of the studies reported no effects of the probiotic intervention on some aspects of task-based fMRI^32,41,42,50,53,55^. On the other hand, some studies reported lower engagement of a variety of brain regions (precuneus, mid and posterior cingulum, hippocampus, parahippocampal gyrus, amygdala, mid and posterior insula, frontal and temporal cortices) during mostly negative emotions, i.e., unpleasant stimuli or angry and fearful faces, after probiotic intervention compared to placebo^31,37,42,50^. In addition, some studies reported lower engagement of brain regions (anterior cingulum, putamen) during neutral emotional stimuli^37,53^. In contrast to the emotional paradigms, the number of cognitive tests was lower, and tests were more heterogeneous in design and resulted in less prominent probiotic effects on brain function.

**Table 4:** Tasks performed during functional magnetic resonance imaging (fMRI) and reported probiotic effects

Study	fMRI paradigm	Measured parameter and direction of effect	Localisation of effect
Tillisch et al.^31^	Emotional faces attention task (angry and fearful faces (ME), control: geometric forms (MF))	Brain activity, Probiotics < placebo	ME > MF: primary viscerosensory, somatosensory cortex (posterior and mid insula) (with a priori-defined region of interest analysis based on a network with altered connectivity, while no effect with a whole-brain analysis)
		Task-related functional connectivity, sig. differences post vs pre across all groups, post hoc: Probiotics 4 weeks < baseline, (placebo 4 weeks = baseline)	PLS of all ME > MF: Widely distributed network of primary interoceptive and somatosensory regions, midbrain cluster centred around periaqueductal grey, prefrontal cortex, precuneus, basal ganglia, parahippocampal gyrus
Pinto-Sanchez et al.^50^	Fearful face backward masking paradigm (fearful and neutral faces, control: fixation cross) Whole-brain & a priori selected regions of interest: bilateral amygdala	Brain activity, Probiotics vs placebo	No significant difference between fearful and neutral faces.
		Brain activity, Probiotics < placebo	Fearful faces vs fixation cross: amygdala and frontal and temporal cortices
		Brain activity, Probiotics > placebo	Fearful faces vs fixation cross: occipital regions
Bagga et al.^37^	Emotional-decision making task (neutral (N), unpleasant (U), control: geometric (B) pictures)	Brain activity, Probiotics < placebo	N > B: no sig differences, U > B: precuneus, mid cingulum, parahippocampal gyrus
	Emotional recognition memory task (neutral (N), unpleasant (U), control: geometric (B) pictures)	Brain activity, Probiotics < placebo	N > B: anterior cingulum; U > B: posterior cingulum
Papalini et al.^32^	Emotional face-matching paradigm (angry and fearful faces, control: geometric shapes)	Brain activity, Probiotics vs placebo	No effect
	Emotional face-word stroop paradigm (happy and fearful)	Brain activity, Probiotics vs placebo	No effect
	Classical colour-word Stroop task	Brain activity, Probiotics vs placebo	No effect
Edebol-Carlman et al.^41^	Montreal Imaging Stress Task (stress, control, baseline condition)	Brain activity, Probiotics > placebo	Stress>control*: A40rv, rostroventral area 40, A37dl, dorsolateral area 37 (*not significant after Bonferroni or FDR correction)
		Task-related functional connectivity, Probiotics > placebo	RRC of all stress>control: A4ul, area 4 (upper limbregion) with A37mv, medioventral area37
Rode et al.^42^	Emotional Attention Task (angry and fearful faces (ME), control: geometric shapes (MS))	Brain activity, Probiotics > placebo	ME > MS*: medial area 10, orbital gyrus – orbital area 12/47, lateral area 12/47 (*not significant after Bonferroni or FDR correction)
		Task-related functional connectivity, Probiotics < placebo	RRC of all ME > MS: frontal pole with caudal area 45, Caudal ventrolateral area 6 with occipital polar cortex, frontal pole with inferior frontal junction and sulcus, left with right caudoposterior superior temporal sulcus
Schaub et al.^53^	Emotional task to indicate gender (neutral, semi-fearful, fearful faces, control: fixation cross)	Brain activity, Probiotics vs placebo	No effect
		Brain activity, Probiotics: 31 days < baseline, placebo: no change compared to baseline	Neutral faces vs control: bilateral putamen (Semi-)fearful faces vs control: no effect
Schneider et al.^55^	Working memory task (n-back)	Brain activity, sig. intervention group*time interaction, post hoc Probiotics: 31 days < baseline, placebo: 31 days > baseline	0-back, 2-back: left hippocampus, 1-back: no effect (with a priori-defined region of interest analysis, while no effect with a whole-brain analysis)

*PLS* Partial least squares, *ROI* Region of interest, *RRC* ROI-to-ROI connectivity. If not stated otherwise, the information provided by the respective authors lets us conclude that whole-brain analyses were performed.

Correlation between fMRI outcomes. Tillisch et al. analysed probiotic effects on rsfMRI originating from midbrain regions, amongst others involved in perception and emotional regulation, that showed altered connectivity during an emotional faces attention task (periaqueductal grey, somatosensory cortex, and insula, Table 3 & 4)^31^. The probiotic-induced resting state network correlated negatively with task-induced seed regional brain activity in the probiotic group, which was less strongly correlated in the placebo group^31^. Additionally, the detected probiotic-induced resting state network correlated positively with task-induced regional connectivity^31^.

Cerebral blood flow. One study assessed cerebral blood perfusion (by arterial spin labelling [ASL] MRI), which was not affected significantly (intervention group*time effect) by probiotic intervention, despite group and time effects^54^.

Resting state functional near-infrared spectroscopy (fNIRS). In a healthy study population, Mutoh et al. employed fNIRS in order to assess brain activity before (during screening and at baseline) and after a six-week intervention with *B. breve* M-16 V^44^. Prefrontal cortex laterality index (i.e., left-right difference in brain activity) at rest (3 minutes, sitting) did not differ significantly between the interventions when assessing the study population as a whole^44^. However, when analysing the subgroups with high anxiety state and trait scores, respectively, prefrontal laterality index at rest was significantly lower post-probiotic intervention compared to placebo^44^.

Task-based (fNIRS). In the same study by Mutoh et al., prefrontal cortex laterality index during stress evoked by an arithmetic task was not significantly affected by the probiotic intervention (overall and subgroup analyses)^44^.

Resting state magnetoencephalography (MEG). In MEG and electroencephalography (EEG), the frequency bands beta (13–30 Hz; normal consciousness, active concentration), alpha (8–13 Hz; wakefulness, passive attention, relaxation), theta (4–8 Hz; drowsiness, early states of sleep), and delta (0–4 Hz; deep sleep) are utilised to assess brain activity^58^. Wang et al. assessed the effects of a four-week intervention with *B. longum* 1714 on resting state brain activity by MEG (5 min, eyes closed)^39^. The probiotic intervention resulted in higher theta band power in the bilateral inferior, middle, and superior frontal cortex and the bilateral anterior and middle cingulate cortex compared to placebo^39^. In addition, the probiotic group exhibited lower beta-2 band power in the bilateral fusiform gyrus, bilateral hippocampus, left inferior and superior temporal and bilateral middle temporal cortex, and the left cerebellum compared to placebo^39^. The authors concluded that the probiotic intervention resulted in changes in resting state brain activity associated with increased vitality and stress-related neural responses^39^.

Task-based MEG. In the same study by Wang et al., utilising a “Cyberball game” as a social stress paradigm, probiotic intake compared to placebo resulted in higher theta band power in one cluster, which included the right inferior, bilateral middle, and superior frontal cortex, left anterior and bilateral middle cingulate cortex, and the right supramarginal gyrus^39^. Moreover, the probiotic intervention also presented higher alpha band power in one cluster, including the right inferior, bilateral middle, and superior frontal cortex, the bilateral anterior and middle cingulate cortex, and the right supramarginal gyrus in both conditions (in- and exclusion) of the paradigm, regions involved in the neural processing of social stress^39^. However, no significant time*group interaction effects were observed^39^.

Resting state electroencephalography (EEG). Unlike MEG, which is utilised to record the brain’s magnetic fields, EEG is utilised to record the brain’s electrical fields^59^. Two studies performed resting state EEG to assess probiotic effects on brain activity (Table 5). In healthy male subjects, Kelly et al. observed lower F3 (placement of the electrode at frontal location 3) zero crossings, second derivative (a measurement that encodes wave frequency; a reduced number of zero crossings indicates fewer high-frequency components at this recording location) post eight-week intervention with *L. rhamnosus* JB-1 vs placebo, but not compared to baseline^34^. Kikuchi-Hayakawa et al. found significantly lower theta power, but no significant differences in the alpha or beta power, or beta/alpha ratio between a four-week intervention with *L. casei* Shirota and placebo, in subjects with sleeping problems^47^.

**Table 5:** Resting and automated electroencephalography (EEG) and reported probiotic effects

Study	EEG paradigm	Measured parameter and effect, probiotic vs placebo
Kelly et al.^34^	Resting EEG (eyes closed)	Lower F3 zero crossings, second derivative. No effect on delta, theta 2, alpha 1, alpha 2, or beta waves. No effect on zero crossings, first derivative. (Several differences were observed after both interventions compared to baseline.)
Kikuchi-Hayakawa et al.^47^	Resting EEG (eyes open and eyes closed, 2 min each)	Lower theta power in the resting state, eyes open. No effect on alpha, beta, or alpha/beta ratio in resting state eyes opened or on any wave power with eyes closed.
Malaguarnera et al.^49^	Automated EEG	No effect

Automated EEG. Malaguarnera et al. performed a 90-day parallel intervention with *B. longum* plus fructo-oligosaccharides in cirrhotic patients and reported no differences in automated EEG (resting, at 30, 60, 90, 120 days) between the probiotic group compared to placebo or compared to baseline within groups^49^ (Table 5).

Task-based EEG. In addition to utilising EEG during rest, EEG has also been employed to measure brain activity during task performance (Table 6). Adikari et al. conducted an eight-week intervention to assess the effects of *L. casei* Shirota on anxiety-induced physiological parameters in competitive football players, using the digit vigilance test, a cognitive task^35^. In pairwise comparisons, the probiotic group exhibited significantly higher absolute theta and delta brain wave power, brain waves associated with sleep and relaxed state, but not alpha, beta or gamma brain wave power, compared to placebo at week four but not week eight^35^. In addition, Kikuchi-Hayakawa et al. investigated the effects of a four-week intervention with *L. casei* Shirota on brain activity in the auditory oddball task^47^. They found no differences for alpha or beta power, or beta/alpha ratio but did observe significantly lower theta power during the task in the probiotic group in the afternoon^47^. Furthermore, Li et al. conducted a 30-day intervention with *P. acidilactici* in patients with MDD on active antidepressant treatment plans to examine the effects of the probotic intervention on brain activity during the “Doors Guessing Task”, a paradigm to assess reward processing^56^. Stimulus-preceding negativity waveform amplitude (an indication of award anticipation) was significantly greater in the probiotic group compared to placebo post-intervention^56^. Furthemore, at post-intervention, stimulus-preceding negativing waveform amplitude was increased compared to baseline in the probiotic group, with no significant changes in the placebo group^56^.

**Table 6:** Task-based electroencephalography (EEG) and reported probiotic effects

Study	EEG paradigm	Measured parameter and effect, probiotic vs placebo
Adikari et al.^35^	EEG recorded during the digit vigilance test	Higher theta and delta brain waves. No effect on alpha, beta, or gamma waves. No statistically significant time*group interaction effects.
Kikuchi-Hayakawa et al.^47^	EEG recorded during the auditory oddball task	Lower theta power. No effect on alpha, beta, or alpha/beta ratio during the task.
Li et al.^56^	EEG recorded during “Doors Guessing Task”, a paradigm to assess reward expectation and consumption	The stimulus-preceding negativity waveform amplitude was significantly greater post-intervention. No effect on feedback-related negativity waveform amplitude.

Sleep EEG. Three studies assessed sleep quality by objective measures using EEG (Table 7). Takada et al. investigated whether an intervention with *L. casei* Shirota improved sleep quality in medical students subjected to academic stress, utilising overnight single-channel EEG recordings^36^. Probiotic intake prevented a reduction in N3 sleep (deep sleep) as the exam approached compared to placebo and increased delta power during the first sleep cycle (measured as index of sleep intensity) of more than 20% as the exam approached^36^. All mentioned parameters were reported as significant as time*group interaction effect^36^.

**Table 7:** Sleep electroencephalography (EEG) studies and reported probiotic effects

Study	Measured parameter and effect, probiotic vs placebo	Other sleep outcomes*
Takada et al. ^36^	Maintained N3 sleep, significant at week 9. Increased delta power as the exam approached during the first sleep cycle.	SOL, TST, SE, % WASO
Nakagawa et al.^45^	Did not report on brain waves.	SPT, TST, SE, WASO, rate of wake time after sleep onset, SOL, REM latency, time in bed
Ho et al.^46^	Higher N3%. No effect on relative values of beta, alpha, theta, or delta waves in overall sleep. Lower theta power % on day 15 when overall sleep was divided into sleep stages.	TST, SOL, WASO, SE, number of awakenings, arousal index

*N3* stage 3 non-REM sleep (deep sleep), *REM* rapid eye movement, *SE* sleep efficiency, *SOL* sleep onset latency, *SPT* sleep period time, *TST* total sleep time, *WASO* wakefulness after sleep onset. *outside the scope of this systematic review and hence not reported in greater detail.

In individuals with sleep problems, Nakagawa et al. conducted a four-week intervention to assess the effects of *L. helveticus* MIKI-020, its fermentation products, and theanine^45^. Sleep EEG was assessed for three days before and at the end of intervention; however, brain waves were not reported^45^. Similarly, Ho et al. examined the effects of an intervention with *L. plantarum* PS128 on sleep quality by a miniature polysomnography^46^. N3% (percentage of deep sleep out of total sleep) of the probiotic group was significantly lower compared to placebo^46^. Alpha, beta and delta waves were not significantly affected^46^. Not when assessed as a whole, but when overall sleep was broken down into sleep stages (N1, N2, N3, and REM), they found that theta power % during N1 (light sleep stage that occurs right after sleep onset) of the probiotic group on day 15 (mid-study) was significantly lower compared to placebo^46^. There were no significant differences when considering N2, N3, or REM^46^.

### Brain tissue metabolite concentrations

Proton magnetic resonance spectroscopy outcomes (MRS). In post-COVID-19 ME/CFS patients, a study found significant time*group interaction effects indicating increased creatine (free creatine and phosphocreatine) levels in left frontal white and grey matter and increased choline (free choline, glycerophosphocholine and phosphorylcholine) levels in the thalamus, in favour of the probiotic intervention over the placebo^52^. While choline levels were not affected by any intervention compared to baseline, creatine and N-acetyl aspartate levels increased significantly compared to baseline, mainly in the probiotic group, in some parts of the thalamus, frontal, precentral, paracentral, and parietal white and grey matter^52^.

### Correlation of brain imaging or electrophysiology results with other parameters

Although out of the immediate scope of this systematic review, the included studies’ attempts to understand the modes of action and clinical outcomes of probiotic-mediated effects on brain structure and function are worth mentioning. The following non-imaging outcomes were assessed: general health, mood, emotional regulation, stress, cognition and memory, sleep quality, gastrointestinal health, immune response, signalling molecules, faecal microbiota and metabolites, heart rate (variability), and skin conductance.

Probiotic intervention-related correlations between brain imaging or electrophysiology results and other parameters were reported on in seven of the 19 studies. The remaining studies either did not assess correlations or did not report the results of such analyses.

The majority of studies assessed correlations between outcomes on emotional regulation, depression and anxiety. In one of those studies, after a four-week intervention with a multi-strain probiotic product in healthy subjects, Bagga et al. observed that brain activity in the cerebellum and cingulum during an emotional recognition task (with neutral and unpleasant stimuli) correlated negatively with improved general well-being in the probiotic but not the placebo group^37^. Similarly, the study by Pinto-Sanchez et al. found that after six-week intervention with *B. longum* in IBS patients, decreased engagement of the amygdala during a negative emotional task (fearful faces) correlated with lowered depression scores in the probiotic, but not the placebo group^50^. In the probiotic group, the reduced engagement of the amygdala was also found more frequently in IBS patients experiencing relief of gastrointestinal symptoms following the intervention^50^. Contrary, Schaub et al. could not find correlations between brain activity during a negative emotional task (fearful faces) and depression or anxiety ratings after a four-week adjunctive multi-strain probiotic intervention in depressed patients on active anti-depressant medication plans^53^. In the same depressed study population, Yamanbaeva et al. found probiotics-induced changes in structural connectivity (fractional anisotropy and mean diffusivity) in the left uncinate fasciculus and decreased functional resting state connectivity between amygdala and superior parietal lobule that correlated with improved depression scores in the probiotic, but not placebo group^54^. No correlation between voxel-based morphometry outcomes and psychological symptoms was observed^53^.

Li et al. also performed some correlations to explore the relationship between subjective and objective indicators of depression and anhedonia^56^. They observed a significant negative correlation between changes in stimulus-preceding negativity waveform amplitude and changes in anxiety and depression scores in the probiotic group. They also observed a weak positive correlation between changes in stimulus-preceding negativity waveform amplitude and changes in anhedonia scores^56^. Furthermore, after a four-week intervention with *B. longum* 1714 in healthy subjects, energy and vitality ratings were positively correlated with changes in averaged theta band power and were negatively correlated with changes in beta-3 band power during resting state, solely in the probiotic group^39^.

Other studies assessed correlations between aspects of cognition, especially working memory. For instance, Schneider et al. found hippocampal activation to be correlated with reaction time during a working memory task (n-back task), with inverse directions after probiotics vs placebo intervention in a depressed patient cohort and significantly different correlations between the groups^55^. In terms of signalling molecules, they found no correlation between brain activation in the memory task and the levels of brain-derived neurotrophic factor^55^. Another study found a negative correlation between stress-induced working memory changes (assessed by digit span backward scores after socially-evaluated cold pressor test) and brain activity in bilateral inferior prefrontal cortex during cognitive control (by colour-word Stroop task) in the probiotics but not the placebo group^32^. This correlation also differed significantly between groups for the right interior prefrontal cortex^32^. Brain activity in the dorsolateral, dorsomedial and ventrolateral prefrontal cortex was activated during the cognitive control task, and probiotics-induced changes in faecal microbiota composition (especially increased relative abundance of the Ruminococcaceae_UCG-003 genus) did not correlate significantly^33^. In addition, brain activity during an emotional task did not correlate with changes in microbiota composition^53^. In the IBS study^50^, higher faecal probiotic abundance (assessed by quantitative polymerase chain reaction, qPCR) was significantly correlated with decreased amygdala activation during the negative emotional task^51^. Faecal probiotic abundance also correlated with increased plasma butyric acid concentrations, which in turn correlated with decreased amygdala activation, as well as decreased anxiety and depression scores^51^. Furthermore, increased plasma concentrations of tryptophan, N-acetyl tryptophan, and pentadecanoic acid correlated significantly with decreased amygdala activation, in the entire study population and also when assessing the probiotic group, but not the placebo group, separately^51^. Other glycine-conjugated bile acids or free fatty acids did not correlate with the fMRI findings^51^.

Although some of the above-mentioned correlations include parameters with potential stress components, only one study assessed correlations on direct stress-related outcomes. As such, Wang et al. found that *B. longum* 1714-induced changes during a social stressor (cyberball game, exclusion condition) in neural activity, i.e., theta and alpha band power, correlated positively with changes in feelings of belonging, self-esteem, control, and meaningful existence, but not after placebo^39^. According to the methods section, they also assessed correlations to other psychological ratings, but it seems that those were not significant (since not reported in the results section)^39^.

One of the sleep EEG studies, Ho et al., found moderate to high correlations between the changes in alpha, beta and delta power % and depression scores in participants with insomnia after 30 days of supplementation with *L. plantarum* PS128^46^.

## Discussion

To the best of our knowledge, this is the first systematic review to exhaustively report on the effects of probiotic intake on human brain health utilising investigations with brain imaging or electrophysiology outcomes. Interestingly, despite a relatively small number of studies and considerable heterogeneity between the study protocols in terms of study populations, probiotic interventions, and brain outcome assessment techniques and analyses thereof, we could identify a number of consistent results. Collectively, probiotic intake appears to modify resting state brain activity and functional connectivity, decrease the involvement of several brain areas, especially during negative emotions, and improve sleep quality.

A fundamental strength of this systematic review is its focus on objective multimodal brain outcome assessment techniques. So far, solely some of the findings from six studies obtained by functional magnetic resonance imaging had been summarised^60^.

Considering the examined effects, task-based fMRI effects seem to occur most often in response to negative emotions. Although most of these studies focus on mood, the applied emotional tasks often have a cognitive component^61,62^. The heterogeneity in task-based outcomes might likely arise from methodological differences in the variety of tasks used and data analyses applied, despite all paradigms being well-validated. Furthermore, results may be domain-specific.

**Fig. 1:**
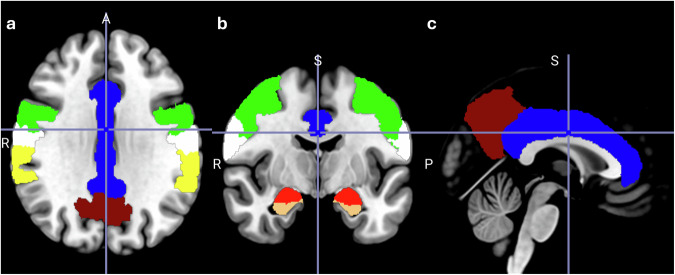
Brain regions most commonly affected by probiotic interventions. Regions are presented on a standard brain in **a** axial, **b** coronal, **c** sagittal view. Probiotics most often affected the highlighted brain regions: hippocampus (orange), cingulate gyrus (blue), supramarginal gyrus (yellow), precentral gyrus (green), postcentral gyrus (white), amygdala (red), precuneus (brown) - across all studies, independent of paradigm applied, technology used or analyses performed.

Across all studies, no matter the paradigm or analyses performed, probiotics seem to affect most often cingulate regions, hippocampal regions and supramarginal gyrus, with involvement in at least six comparisons (probiotic vs placebo). Additionally, exclusively in the fMRI analyses, precuneus, pre- and postcentral gyrus and amygdala were involved in four comparisons (probiotic vs placebo). Generally, frontal regions seem to be affected quite often in the studies included in this review. The regions that are most commonly affected by probiotic interventions are visualised in Fig. 1. Of note, since the majority of studies applied whole-brain analyses approaches (Supplementary Table 9), it can be assumed that probiotic interventions generally evoked stronger signal changes in those reported brain regions. Probiotic-induced changes in the included EEG and MEG studies, independent of whether they were conducted during rest, sleep or during task performance, most often showed an impact on theta brain waves, which are indicative of early stages of sleep or drowsiness. Interestingly, the combined findings of this review are consistent with the results of studies that correlate brain connectivity and gut microbiota composition^63,64^. In a systematic review of 16 such studies, the authors found associations between brain connectivity in the salience (most prominently the insula and cingulate cortex), default mode, and frontoparietal networks and gut microbiota composition and diversity, with low specificity likely due to the heterogeneity of the included studies^65^. Our systematic review on probiotics’ effects as a potential gut microbiome modulating intervention now adds towards the understanding of directionality and causality.

Although rsfMRI changes are conceptually predictive of brain responses to specific tasks, only Tillisch et al.^31^ investigated this in the context of probiotics and negative emotional responsiveness. With resting state conditions being much less demanding than the application of tasks in the brain imaging environment, further investment in such correlative analyses may facilitate the use of especially rsfMRI as a non-invasive surrogate marker for brain and mental health effects of probiotics and simultaneously allow assessment of larger cohorts and/or compromised subjects.

The effects of probiotic intake on brain morphometry and structural connectivity (with alterations traditionally being associated with various diseases) were inconclusive and limited to a few parameters.

Previous studies have established that probiotic supplementation may impact subjectively assessed clinical symptom ratings^24–26^ but correlating these findings to objective measures, such as brain imaging or electrophysiology outcomes, allows speculations regarding the biological mechanisms and clinical relevance of these effects. Here, the reported correlations are mostly based on fMRI outcomes and measures of mood, especially depression. For example, lower brain activity during negative emotional stimulation was associated with improved mood and well-being in healthy individuals^37^ and IBS patients^50^ after probiotic but not placebo intake. Although not assessing correlations specifically, other studies in healthy cohorts allow similar interpretations regarding emotional regulation^42,43^ and cognition^66^ (the latter was excluded from this systematic review due to its non-randomised design). In depressed patients on active antidepressant treatment, changes in depressive symptom scores did not correlate with brain function during negative emotional stimulation^53^, but with functional and structural connectivity during resting state^54^. Not surprisingly, improved depression scores correlated with improved sleep patterns^46^. Furthermore, increases in waveform amplitude (measured by EEG) related to reward anticipation were negatively correlated with improved depression and anxiety scores^56^. The latter two, amongst the otherwise rather concise EEG studies, are the only ones reporting correlations, while most MRI studies, especially the more recent ones, employed a comprehensive design with a broad spectrum of outcome measures.

Hitherto, most studies assessed probiotic effects on brain health in healthy young-to-middle-aged populations. This may have been the case as studies in these populations offer easier recruitment and lower safety risks, as well as a more homogenous study group. Notably, several studies observed probiotic effects in healthy populations, supporting that the gut-brain axis is modifiable despite an expected ceiling effect with limited room for improvement. Yet, the generalisability of the results is only applicable to a healthy adult population. Ascone et al. argued for the use of a young adult population because of expected higher neuronal plasticity and hence a facilitated detection of any effects^40^. However, they reported one of the few studies with non-differential brain structure and function post-probiotic compared to placebo. Positive results in a healthy population related to decreased stress, improved sleep, or improved emotional regulation may be of great value for optimising health as a preventative measure. The current evidence, on the brain imaging or electrophysiology level, does not allow conclusions on whether probiotics are more effective in patient cohorts than in healthy subjects, but such a tendency is indicated on the level of symptomatology^67^. Hence, we call for more studies in compromised populations, including those with (subclinical) cognitive impairment, e.g., in an ageing population, or elevated stress levels (as e.g., mimicked by those studies testing cognition when buffered against stress^32^ or sleep in the setting of academic examination stress^36^) to confirm the subtle changes observed in mostly healthy cohorts. Importantly, to correct for factors leading to heterogeneity, larger sample sizes may be needed when studying patient cohorts. Finally, it is also time to conduct probiotic interventions with brain imaging or electrophysiology outcomes in conditions of disturbed gut-brain interactions, with IBS^50^ and depressed patients^53–56^ as pioneering examples. In IBS patients with comorbid depression and/or anxiety symptoms, Pinto-Sanchez et al. observed probiotic-induced changes in brain regions (previously associated with depression and anti-depressant treatment) whose activity correlated with gastrointestinal symptom scores^50^. Probiotic effects on major depression have so far been studied as an adjunctive to antidepressant treatment using brain imaging techniques^53–56^. The positive effect of probiotics on depressive symptomatology is moreover supported by a recent meta-analysis^26^. In children diagnosed with autism spectrum disorder, six-month multi-strain probiotic intake modified brain activity (as assessed by EEG) in conjunction with improved behavioural measures compared to placebo^68^. To note is that all included studies relied on randomisation, and none utilised typical covariates such as age, sex and others in their analyses.

The studies included in this review investigated a diversity of probiotics, which may contribute to observed potential probiotic effects on a broad spectrum. Mainly, probiotics of the *Bifidobacterium* genus and *Lactobacillaceae* family were able to modulate brain function. The diversity of results, however, does not enable us to draw conclusions on which probiotic species, strain or mixture may be the most effective. Interestingly, all EEG, MEG and fNIRS studies investigated single-strain products, while all rsfMRI and most task-based fMRI studies used multi-strain probiotics. All studies with multi-strain probiotic products, except one, had an intervention period of four weeks, whereas the intervention period for single-strain products ranged from four to 28 weeks. The optimal intervention length of probiotic interventions is not known. Schaub et al. motivate the duration of their intervention (31 days) with observed effects on behavioural outcomes^53^. Furthermore, Kelly et al. argue that anti-depressants do not have a strong effect in healthy subjects, plus they do need some onset time of approximately four weeks until effective, and the same might be applied regarding probiotic effects on brain function^34^. While four-week probiotic interventions have been shown to be sufficient to simultaneously modify the gut microbiota and brain function^32,33^, a change of the microbiota itself might not be necessary to see effects on brain health, as heat-inactivated *L. gasseri* has been shown to be effective in modulating brain function, as assessed by sleep EEG^69,70^ similar to the probiotics summarised in this review.

In/ex vivo studies investigating the underlying mechanisms could aid in understanding probiotic effects on the gut-brain axis beyond direct microbiota modulation. An interesting, but so far missing approach, is the identification of neurotransmitter levels by, e.g., magnetic resonance spectroscopy. The first approaches to assess brain tissue metabolites^52^ or blood metabolites^51^ are interesting and will provide invaluable information for the field. Furthermore, implementing follow-up of participants (as piloted by one of the EEG studies^49^), weeks after intervention, would be interesting to evaluate if probiotic effects can be long-lasting or how quickly they are declining. Also, the field is thus far lacking dose-response studies, which would be of great interest.

Several study products contained additional ingredients, including fructo-oligosaccharides^49,52^ and theanine^45^, with the potential to influence the gut microbiome, CNS, or immune system. In these cases, it is not possible to attribute the potential effects specifically to the probiotic compounds. However, this was not always transparently disclosed by the authors. Moreover, the importance of identical placebo and test products should be obvious, but this was not implemented by some of the investigated studies. Mental health research is susceptible to a strong placebo effect, which emphasises the importance of selecting an appropriate placebo even when applying objective brain outcome assessment methods^71^.

When studying probiotics using brain imaging or electrophysiology techniques, the absence of standardised procedures predominantly hampers our ability to draw conclusions from the cumulative evidence. However, human behaviour is formed by coordinated activities from large-scale networks, and studies of how the whole brain interplays are of interest^72^. The latter is likely one of the reasons why probiotic-induced alterations of brain function point towards similar conclusions. Studies were usually powered for their respective primary outcome, and as that is rarely a pure brain imaging or electrophysiology outcome, these investigations may often be underpowered.

We believe that this systematic review raises awareness and provides a summary that will be important for shaping protocols (for recommendations see Table 8) for this relatively new research field. Future probiotic and gut-brain axis research, using brain outcome assessment techniques in combination with in/ex vivo mechanistical assessments, could help clarify possible effects of probiotics on brain function beyond the potential preventive effects, inform clinical use and formulation of targeted products and interventions.

**Table 8:** Recommendations for planning probiotic interventions to impact brain outcomes

Category	Recommendation
Target population	As the majority of included studies observed subtle effects in mostly healthy cohorts, we recommend more studies in compromised populations. For such heterogeneous populations, a larger sample size is warranted.
Selection of brain outcomes	Our systematic review confirms the use of brain imaging and electrophysiology as beneficial tools to assess the effects of probiotic interventions on the brain. Furthermore, several studies support the use of resting state functional magnetic resonance imaging (rsfMRI) and task-based functional magnetic resonance imaging (fMRI) protocols investigating the response to negative emotions, as well as sleep electroencephalography (EEG).
Probiotic strain selection	As so many different probiotic strains were utilised, we are unable to recommend the ideal strain or comment on the effectiveness of single- vs multi-strain products.
Probiotic dosage	Conducting dose-response studies would be invaluable.
Intervention duration	The ideal intervention length to assess the effects of probiotics on brain outcomes is not clearly established (ranging from 4–28 weeks). Thus, studies to investigate and compare multiple intervention lengths are necessary for the field. Furthermore, studies to investigate the effects of probiotics weeks to months after intervention cessation are also necessary.
Selection of study product ingredients	Consider probiotic study products that do not contain additional ingredients that influence the gut-brain axis. Furthermore, it is important for the control/placebo products to be identical to the active products (minus the addition of the probiotic strains).
Covariates	It is important for probiotic interventions to consider covariates such as sex, age, baseline health status, baseline cognition/mental health, etc. Such may be controlled for during the randomisation or analysis phase, amongst others.
Transparent reporting	We recommend more detailed descriptions of methodologies (blinding procedures, ingredients in study products, brain outcome analyses pipelines, etc).

## Methods

### Protocols and registration

This systematic review was conducted following PRISMA guidelines, and initially submitted to PROSPERO on June 21, 2023. The registration record was automatically published after basic automated checks for eligibility and formally registered on July 2, 2023 under ID CRD42023438493.

### Information sources and search strategy

A medical research librarian at Örebro University conducted a search in the following databases: MEDLINE, Embase, and Web of Science. The basic structure of the search string was as follows: Probiotics AND (neuroimaging OR (imaging techniques AND brain) OR (electrophysiology techniques AND brain)). Relevant synonyms and subject headings for the search terms were used. The search was restricted to articles published in English. Conference abstracts were excluded from the Embase results. The complete search strategy can be found in Supplementary Note 1. The original and updated searches were conducted on June 20, 2023, January 9, 2024, and August 5, 2025, respectively. Search updates were performed without date restriction, and automatic duplicate removal was mainly handled by the Covidence tool. In addition to the described search strategy, a targeted search was performed in the clinical trials registers and references of the included studies to ensure that additional studies meeting the search criteria were not overlooked. In addition, the scientific network of the authors was consulted.

### Eligibility criteria

Eligible full-text articles met the following criteria: randomised, placebo-controlled trials, participants aged 18 years and older, oral probiotic interventions (longer than one week; alone or in combination with other dietary interventions, e.g., prebiotics), and outcomes assessed with brain imaging or electrophysiology techniques.

### Data management

The Covidence software was utilised for study selection, data extraction, and risk of bias and study quality assessment.

### Study selection

Two authors (J.R., A.H.) independently screened all titles and abstracts of unique records, with discrepancies discussed among a team of three authors (J.R., A.H., and R.F.). The full text screening of eligible articles was performed by three authors (J.R., A.H., R.F.), with each article evaluated by at least two authors and discrepancies discussed among all three.

### Data extraction

All authors participated in the data extraction and quality assessment, with at least two authors per included article and discrepancies were solved through discussion. The articles authored by J.R., A.H., and J.K. were extracted and evaluated by J.P.G.M. and R.F. to decrease biases. When the extraction matrix was complete for all included papers, the results were discussed between all authors, discrepancies identified and resolved.

### Reporting of results

In accordance with the scope of this systematic review, all reported results focus on the comparisons between the probiotic and placebo intervention, despite some studies including other intervention groups (e.g., no-intervention controls) or assessing differences in population characteristics as well as data on experiment validation.

### Risk of bias and study quality assessment

The quality of the included studies was assessed with Covidence, in accordance with Higgins et al. in determining the scoring strategy^73^. Study quality of the individual studies was scored as Low (high risk of bias), High (low risk of bias), or Unclear using the following parameters: sequence generation, allocation concealment, blinding of participants and personnel, blinding of outcome assessment, incomplete outcome data, selective reporting, and other sources of bias (including publication bias and commercial interests). Assessments were performed for each study by two authors of the study team, and discrepancies were resolved via discussion with the entire team. To further address potential publication bias, with e.g., negative findings not being reported/published due to commercial interests (potential funding bias), and since the variable outcome measures did not allow for traditional funnel plot estimations, we searched the database Clinicaltrials.gov for “probiotic” AND “brain” and screened the records in March 2024 and updated in August 2025. The results of these assessments of risk of bias and study quality can be found in Supplementary Notes 3 and 7, Supplementary Fig. 4, Supplementary Tables 5 and 6. References ^74–77,78–80^ are solely cited in the Supplementary Information.

## Supplementary information

Supplementary information

## Data Availability

This is a systematic review synthesising published work. No data have been generated for this work. All information is to be found in this article and its Supplementary Information file.

## Code availability

No code was developed or used for this work.
