# Supplementary material for: The effects of oral probiotic intervention on brain structure and function in human adults: a systematic review
Source: NPJ Biofilms Microbiomes. 2026 Jan 7;12:6. doi: 10.1038/s41522-025-00872-x (PMC12779965; doi:10.1038/s41522-025-00872-x)
Supplement: Supplementary file 1 — Supplementary information [file 41522_2025_872_MOESM1_ESM.pdf]

Supplementary Information

The effect of oral probiotic intervention on brain structure and function in human adults: a systematic review

Ashley N Hutchinson[1], Amanda E Antonsson[1], Richard A Forsgård[1], Julia König[1], John-Peter Ganda Mall[1], Julia Rode[1,2]

[1] Nutrition-Gut-Brain Interactions Research Centre, School of Medical Sciences, Faculty of Medicine and Health, Örebro University, Örebro, Sweden
[2] School of Health Sciences, Faculty of Medicine and Health, Örebro University, Örebro, Sweden

Correspondence: Julia Rode, Julia.rode@oru.se, 0046 19 30 3000

Supplementary Note 1. Search Strategy

Medline, first run on 2023-06-20, updated on 2024-01-09 and 2025-08-05.

(**Ovid MEDLINE(R) ALL** 1946 to August 05, 2025)

/ =Mesh-term

exp =exploded Mesh-term

ab =abstract

kf =keyword heading word

ti =title

nm =name of substance word

Search terms			Number of results
Probiotics			
	1	exp Probiotics/	
	2	exp Lactobacillus/	
	3	exp Bifidobacterium/	
	4	(probiotic* or psychobiotic* or pro-biotic* or psycho-biotic*).ab,kf,ti.	
	5	(Lactobacill* or "Betabacteri*" or "Lactobacileae" or Lactobacter* or acidophilus* or "enpac" or "lacfer" or "lacteol" or "lactophil" or "Thermobacterium intestinale" or "viacil" or "Bacillus casei g" or "Bacillus g" or "Betabacterium breve" or	

		"Levilactobacillus brevis" or "Bacterium curvatum" or "Bacillus acidificans longissimus" or "Bacillus delbrucki" or "bacillus delbrueckii" or "Bacterium delbrucki" or "Plocamobacterium delbrucki" or "Thermobacterium cereale" or "Ulvina delbrucki" or "Bacillus bulgaricus" or "Bacillus lactis acidi" or "Thermobacterium lactis" or "Bacillus casei e" or "Bacillus e" or "Caseobacterium e" or "Plocamobacterium helveticum" or "thermobacterium helveticum" or "Streptobacterium plantarum").ab,kf,ti,nm.	
	6	(Bifidobacter* or "Actinobacterium bifidum" or "Actinomyces bifidus" or "Actinomyces parabifidus" or "Bacillus bifidus" or "Bacterium bifidum" or "Bacteroides bifidus" or "Bifidibacterium bifidum" or "Cohnistreptothrix bifidus" or "Nocardia bifida" or "Tissieria bifida").ab,kf,ti,nm.	
	7	exp Lacticaseibacillus/	
	8	(Lacticaseibacillus or Lactobacillus casei or "Bacillus a" or "Bacillus casei a" or "Bacterium casei a" or "casei, lactobacillus" or "caseobacterium vulgare" or "lactobacilli casei" or "Lactobacterium casei" or "streptobacterium casei" or "Lactobacillus casei").ab,kf,ti,nm.	
	9	Bacillus/	
	10	Bacillus clausii/ or Bacillus coagulans/ or Bacillus licheniformis/ or Bacillus pumilus/ or Bacillus subtilis/	
	11	(bacillus or "clostridium licheniforme" or "Denitrobacillus licheniformis" or "Bacillus licheniformis" or "b. subtilis" or "Bacillus natto" or "Bacillus subtilis spore" or "Bacillus uniflagellatus" or "Bacterium subtilis" or "Vibrio subtilis" or "Bacillus subtilis" or "Alkalihalobacillus clausii").ab,kf,ti,nm.	
	12	exp Lactococcus/	
	13	(Lactococcus or "Enterococcus seriolicida" or "Enterococcus serolicida" or "Streptococcus garvieae" or "Bacterium lacti*" or "Streptococcus lactis").ab,kf,ti,nm.	
	14	Limosilactobacillus fermentum/	
	15	("Limosilactobacillus fermentum" or "Bacillus casei d" or "Bacillus d" or "fermenti, lactobacillus" or "Lactobacillus cellobiosus" or "lactobacillus fermenti" or "lactobacillus fermentii" or "Lactobacterium fermentum" or "Limosilactobacillus fermentum" or "Lactobacillus fermentum").ab,kf,ti,nm.	

	16	Limosilactobacillus reuteri/	
	17	("Limosilactobacillus reuteri" or "Lactobacillus fermentum reuteri" or "Lactobacillus reuteri").ab,kf,ti,nm.	
	18	Pediococcus pentosaceus/	
	19	"Pediococcus pentosaceus".ab,kf,ti,nm.	
	20	exp Propionibacterium/	
	21	("Propionibacterium" or "bacterium acidi propionici" or "Bacillus acnes" or "Corynebacterium acnes" or "Corynebacterium parvum" or "Corynebacterium parvum infectiosum" or "Cutibacterium acnes" or "P. acnes" or "Propionibacteria acnes" or "Propionicibacterium acnes" or "Propionobacterium acnes" or "Proprionibacterium acnes" or "strain CN 6134" or "Bacterium acidi propionici a" or "Bacterium acidi propionici d" or "Propionicibacterium freudenreichii" or "Propionicibacterium freudenreichii shermanii" or "Bacille granuleux" or "Corynebacterium granulosum" or "Propionicibacterium granulosum").ab,kf,ti,nm.	
	22	exp Saccharomyces/	
	23	(Saccharomyce* or "CBS 5926" or "baker yeast" or "baker`s yeast" or "brewer yeast" or "brewer`s yeast" or "s cerevisiae" or "saccaromyces cerevisiae").ab,kf,ti,nm.	
	24	Streptococcus salivarius/ or Streptococcus thermophilus/	
	25	("Streptococcus salivarius" or "Streptococcus thermophilus").ab,kf,ti,nm.	
	26	"Weizmannia coagulans".ab,kf,ti,nm.	
	27	Bifidus.ab,kf,ti,nm.	
	28	Escherichia coli/	
	29	("Escherichia coli Nissle" or "Escherichia coli strain Nissle").ab,kf,ti,nm.	
	30	or/1-29	
Neuroimaging			
	31	exp Neuroimaging/ or Functional Neuroimaging/ or exp Electroencephalography/ or Magnetoencephalography/	
	32	("neuro imag*" or neuroimag* or "brain activit*" or "brain electrical activity mapping" or "brain cortical thickness" or	

		magnetoencephalography or magneto-encephalography or magnetoencephalogram* or "diffusion tensor imag*" or "diffusion tensor magnetic resonance imaging" or "diffusion tensor mri" or "dti mri" or "diffusion tractograph*" or "functional brain imag*" or "brain mapping" or "brain radiography" or "brain scinitscanning" or neurography or connectome or "dopaminergic mapping" or neuroradiology or "cerebral angiography" or echoencephalography or myelography or pneumoencephalography or eeg or electroencephalogram* or electroencephalography or "brain wave*" or brainwave* or "alpha rhythm" or "beta rhythm" or "delta rhythm" or "gamma rhythm" or "theta rhythm" or "cortical synchronization" or "functional connectivity" or "resting state" or "brain activit*").ti,ab,kf.	
	33	31 or 32	
Imaging techniques			
	34	exp Magnetic Resonance Imaging/ or exp Tomography, Emission-Computed/ or exp positron-emission tomography/ or single-photon/ or exp tomography, x-ray computed/ or spectroscopy, near-infrared/	
	35	("magnetic resonance imaging" or "echo-planar imaging" or "fluorine-19 magnetic resonance imaging" or "magnetic resonance angiography" or "nmr imaging" or "mr tomograph*" or "nmr tomograph*" or "steady state free precession mri" or zeugmatograph* or "chemical shift imaging*" or "chemical shift imaging" or "magnetic resonance image*" or "magnetization transfer contrast imaging" or "mri scan*" or "proton spin tomograph*" or fmri* or "functional mri*" or "functional magnetic resonance imaging" or "nuclear magnetic resonance imaging" or "diffusion weighted imaging" or "echo planar imaging" or "fluid-attenuated inversion recovery imaging" or "functional magnetic resonance imaging" or "multiparametric magnetic resonance imaging" or "T1 weighted imaging" or "T2 weighted imaging" or "emission tomography" or "computer assisted emission tomography" or "photon emission tomography" or "positron emission tomograph*" or "positron-emission tomography imaging" or "pet scan*" or "pet imaging*" or "near-infrared spectroscop*" or "near infrared spectroscop*" or "nir spectroscop*" or "near-infrared spectrometr*" or "near infrared spectrometr*" or "functional near-infrared spectroscopy" or "time resolved near infrared spectroscop*").ti,ab,kf.	
	36	34 or 35	
Brain			

	37	exp Brain/	
	38	(brain or encephal* or prosencephalon or mesencephalon or rhombencephalon or cerebrum or cerebellum or brainstem or pons or cerebra* or intracranial or cerebrovascular or neurovascular or cranial or intracerebral or neuro*).ab,kf,ti.	
	39	37 or 38	
Imaging techniques combined with brain			
	40	36 and 39	
Neuroimaging OR Imaging techniques combined with brain			
	41	33 or 40	
Sets combined			
	42	30 and 41	
Limit to English language			
	43	limit 42 to english language	532

Embase.com, first run on 2023-06-20, updated on 2024-01-09 and 2025-08-05.

/de =Emtree term

/exp =exploded Emtree term

:ti,ab,kw =title, abstract, keyword

Search terms			Number of results
Probiotics			
	1	'probiotic agent'/exp	
	2	'lactobacillus'/exp	
	3	'bifidobacterium'/exp	
	4	probiotic*:ti,ab,kw OR psychobiotic*:ti,ab,kw OR 'pro biotic*':ti,ab,kw OR 'psycho biotic*':ti,ab,kw	
	5	lactobacill*:ti,ab,kw OR 'betabacteri*':ti,ab,kw OR 'lactobacileae':ti,ab,kw OR lactobacter*:ti,ab,kw OR acidophilus*:ti,ab,kw OR 'enpac':ti,ab,kw OR 'lacfer':ti,ab,kw OR 'lacteol':ti,ab,kw OR 'lactophil':ti,ab,kw OR 'thermobacterium intestinale':ti,ab,kw OR 'viacil':ti,ab,kw OR	

		'bacillus casei g':ti,ab,kw OR 'bacillus g':ti,ab,kw OR 'betabacterium breve':ti,ab,kw OR 'levilactobacillus brevis':ti,ab,kw OR 'bacterium curvatum':ti,ab,kw OR 'bacillus acidificans longissimus':ti,ab,kw OR 'bacillus delbrucki':ti,ab,kw OR 'bacillus delbrueckii':ti,ab,kw OR 'bacterium delbrucki':ti,ab,kw OR 'plocamobacterium delbrucki':ti,ab,kw OR 'thermobacterium cereale':ti,ab,kw OR 'ulvina delbrucki':ti,ab,kw OR 'bacillus bulgaricus':ti,ab,kw OR 'bacillus lactis acidi':ti,ab,kw OR 'thermobacterium lactis':ti,ab,kw OR 'bacillus casei e':ti,ab,kw OR 'bacillus e':ti,ab,kw OR 'caseobacterium e':ti,ab,kw OR 'plocamobacterium helveticum':ti,ab,kw OR 'thermobacterium helveticum':ti,ab,kw OR 'streptobacterium plantarum':ti,ab,kw OR bifidobacter*:ti,ab,kw OR 'actinobacterium bifidum':ti,ab,kw OR 'actinomyces bifidus':ti,ab,kw OR 'actinomyces parabifidus':ti,ab,kw OR 'bacillus bifidus':ti,ab,kw OR 'bacterium bifidum':ti,ab,kw OR 'bacteroides bifidus':ti,ab,kw OR 'bifidibacterium bifidum':ti,ab,kw OR 'cohnistreptothrix bifidus':ti,ab,kw OR 'nocardia bifida':ti,ab,kw OR 'tissieria bifida':ti,ab,kw	
	6	bifidobacter*:ti,ab,kw OR 'actinobacterium bifidum':ti,ab,kw OR 'actinomyces bifidus':ti,ab,kw OR 'actinomyces parabifidus':ti,ab,kw OR 'bacillus bifidus':ti,ab,kw OR 'bacterium bifidum':ti,ab,kw OR 'bacteroides bifidus':ti,ab,kw OR 'bifidibacterium bifidum':ti,ab,kw OR 'cohnistreptothrix bifidus':ti,ab,kw OR 'nocardia bifida':ti,ab,kw OR 'tissieria bifida':ti,ab,kw	
	7	'lacticaseibacillus'/exp	
	8	(lacticaseibacillus:ti,ab,kw OR lactobacillus:ti,ab,kw) AND casei:ti,ab,kw OR 'bacillus a':ti,ab,kw OR 'bacillus casei a':ti,ab,kw OR 'bacterium casei a':ti,ab,kw OR 'casei, lactobacillus':ti,ab,kw OR 'caseobacterium vulgare':ti,ab,kw OR 'lactobacilli casei':ti,ab,kw OR 'lactobacterium casei':ti,ab,kw OR 'streptobacterium casei':ti,ab,kw OR 'lactobacillus casei':ti,ab,kw	
	9	'bacillus'/de	
	10	'bacillus clausii'/de OR 'bacillus coagulans'/de OR 'bacillus licheniformis'/de OR 'bacillus pumilus'/de OR 'bacillus subtilis'/exp	
	11	bacillus:ti,ab,kw OR 'clostridium licheniforme':ti,ab,kw OR 'denitrobacillus licheniformis':ti,ab,kw OR 'bacillus licheniformis':ti,ab,kw OR 'b. subtilis':ti,ab,kw OR 'bacillus natto':ti,ab,kw OR 'bacillus subtilis spore':ti,ab,kw OR 'bacillus	

		uniflagellatus':ti,ab,kw OR 'bacterium subtilis':ti,ab,kw OR 'vibrio subtilis':ti,ab,kw OR 'bacillus subtilis':ti,ab,kw OR 'alkalihalobacillus clausii':ti,ab,kw	
	12	'lactococcus'/exp	
	13	lactococcus:ti,ab,kw OR 'enterococcus seriolicida':ti,ab,kw OR 'enterococcus serolicida':ti,ab,kw OR 'streptococcus garvieae':ti,ab,kw OR 'bacterium lacti*':ti,ab,kw OR 'streptococcus lactis':ti,ab,kw	
	14	'lactobacillus fermentum'/de	
	15	'limosilactobacillus fermentum':ti,ab,kw AND 'bacillus casei d':ti,ab,kw OR 'bacillus d':ti,ab,kw OR 'fermenti, lactobacillus':ti,ab,kw OR 'lactobacillus cellobiosus':ti,ab,kw OR 'lactobacillus fermenti':ti,ab,kw OR 'lactobacillus fermentii':ti,ab,kw OR 'lactobacterium fermentum':ti,ab,kw OR 'limosilactobacillus fermentum':ti,ab,kw OR 'lactobacillus fermentum':ti,ab,kw	
	16	'lactobacillus reuteri'/de	
	17	'lactobacillus fermentum reuteri':ti,ab,kw OR 'limosilactobacillus reuteri':ti,ab,kw OR 'lactobacillus reuteri':ti,ab,kw	
	18	'pediococcus pentosaceus'/de	
	19	'pediococcus pentosaceus':ti,ab,kw	
	20	'propionibacterium'/exp	
	21	'propionibacterium':ti,ab,kw OR 'bacterium acidi propionici':ti,ab,kw OR 'bacillus acnes':ti,ab,kw OR 'corynebacterium acnes':ti,ab,kw OR 'corynebacterium parvum':ti,ab,kw OR 'corynebacterium parvum infectiosum':ti,ab,kw OR 'cutibacterium acnes':ti,ab,kw OR 'p. acnes':ti,ab,kw OR 'propionibacteria acnes':ti,ab,kw OR 'propionicibacterium acnes':ti,ab,kw OR 'propionobacterium acnes':ti,ab,kw OR 'proprionibacterium acnes':ti,ab,kw OR 'strain cn 6134':ti,ab,kw OR 'bacterium acidi propionici a':ti,ab,kw OR 'bacterium acidi propionici d':ti,ab,kw OR 'propionicibacterium freudenreichii':ti,ab,kw OR 'propionicibacterium freudenreichii shermanii':ti,ab,kw OR 'bacille granuleux':ti,ab,kw OR 'corynebacterium granulosum':ti,ab,kw OR 'propionicibacterium granulosum':ti,ab,kw	
	22	'saccharomyces'/exp	

	23	saccharomyce*:ti,ab,kw OR 'cbs 5926':ti,ab,kw OR 'baker yeast':ti,ab,kw OR 'baker`s yeast':ti,ab,kw OR 'brewer yeast':ti,ab,kw OR 'brewer`s yeast':ti,ab,kw OR 's cerevisiae':ti,ab,kw OR 'saccaromyces cerevisiae':ti,ab,kw	
	24	'streptococcus salivarius'/de OR 'streptococcus thermophilus'/de	
	25	'streptococcus salivarius':ti,ab,kw OR 'streptococcus thermophilus':ti,ab,kw	
	26	'weizmannia coagulans':ti,ab,kw	
	27	bifidus:ti,ab,kw	
	28	'escherichia coli'/de	
	29	'escherichia coli nissle':ti,ab,kw OR 'escherichia coli strain nissle':ti,ab,kw	
	30	'streptococcus salivarius'/de OR 'streptococcus thermophilus'/de	
	31	#1 OR #2 OR #3 OR #4 OR #5 OR #6 OR #7 OR #8 OR #9 OR #10 OR #11 OR #12 OR #13 OR #14 OR #15 OR #16 OR #17 OR #18 OR #19 OR #20 OR #21 OR #22 OR #23 OR #24 OR #25 OR #26 OR #27 OR #28 OR #29 OR #30	
Neuroimaging			
	32	'brain mapping'/exp OR 'brain radiography'/exp OR 'brain scintiscanning'/exp OR 'connectome'/exp OR 'electroencephalography'/exp OR 'neurography'/exp OR 'neuroimaging'/exp OR 'functional neuroimaging'/exp OR 'magnetoencephalography'/de	
	33	'neuro imag*':ti,ab,kw OR neuroimag*:ti,ab,kw OR 'brain activit*':ti,ab,kw OR 'brain electrical activity mapping':ti,ab,kw OR magnetoencephalography:ti,ab,kw OR 'magneto-encephalography':ti,ab,kw OR magnetoencephalogram*:ti,ab,kw OR 'brain cortical thickness':ti,ab,kw OR 'diffusion tensor imag*':ti,ab,kw OR 'diffusion tensor magnetic resonance imaging':ti,ab,kw OR 'diffusion tensor mri':ti,ab,kw OR 'dti mri':ti,ab,kw OR 'diffusion tractograph*':ti,ab,kw OR 'functional brain imag*':ti,ab,kw OR 'brain radiography':ti,ab,kw OR 'brain scinitscanning':ti,ab,kw OR neurography:ti,ab,kw OR connectome:ti,ab,kw OR 'brain mapping':ti,ab,kw OR 'dopaminergic mapping':ti,ab,kw OR neuroradiology:ti,ab,kw OR 'cerebral angiography':ti,ab,kw OR echoencephalography:ti,ab,kw OR electroencephalography:ti,ab,kw OR myelography:ti,ab,kw OR	

		pneumoencephalography:ti,ab,kw OR eeg:ti,ab,kw OR electroencephalogram*:ti,ab,kw OR 'brain wave*':ti,ab,kw OR brainwave*:ti,ab,kw OR 'alpha rhythm':ti,ab,kw OR 'beta rhythm':ti,ab,kw OR 'delta rhythm':ti,ab,kw OR 'gamma rhythm':ti,ab,kw OR 'theta rhythm':ti,ab,kw OR 'cortical synchronization':ti,ab,kw OR 'functional connectivity':ti,ab,kw OR 'resting state':ti,ab,kw	
	34	#32 OR #33	
Imaging techniques			
	35	'nuclear magnetic resonance imaging'/de OR 'diffusion tensor imaging'/de OR 'diffusion weighted imaging'/exp OR 'echo planar imaging'/de OR 'fluid-attenuated inversion recovery imaging'/de OR 'functional magnetic resonance imaging'/de OR 'multiparametric magnetic resonance imaging'/de OR 't1 weighted imaging'/de OR 't2 weighted imaging'/de OR 'computer assisted emission tomography'/exp OR 'near infrared spectroscopy'/de OR 'functional near-infrared spectroscopy'/de OR 'time resolved near infrared spectroscopy'/de	
	36	'magnetic resonance imaging':ti,ab,kw OR 'echo-planar imaging':ti,ab,kw OR 'fluorine-19 magnetic resonance imaging':ti,ab,kw OR 'magnetic resonance angiography':ti,ab,kw OR 'nmr imaging':ti,ab,kw OR 'mr tomograph*':ti,ab,kw OR 'nmr tomograph*':ti,ab,kw OR 'steady state free precession mri':ti,ab,kw OR zeugmatograph*:ti,ab,kw OR 'chemical shift imaging*':ti,ab,kw OR 'chemical shift imaging':ti,ab,kw OR 'magnetic resonance image*':ti,ab,kw OR 'magnetization transfer contrast imaging':ti,ab,kw OR 'mri scan*':ti,ab,kw OR 'proton spin tomograph*':ti,ab,kw OR fmri*:ti,ab,kw OR 'functional mri*':ti,ab,kw OR 'nuclear magnetic resonance imaging':ti,ab,kw OR 'diffusion weighted imaging':ti,ab,kw OR 'echo planar imaging':ti,ab,kw OR 'fluid-attenuated inversion recovery imaging':ti,ab,kw OR 'functional magnetic resonance imaging':ti,ab,kw OR 'multiparametric magnetic resonance imaging':ti,ab,kw OR 't1 weighted imaging':ti,ab,kw OR 't2 weighted imaging':ti,ab,kw OR 'emission tomography':ti,ab,kw OR 'computer assisted emission tomography':ti,ab,kw OR 'photon emission tomography':ti,ab,kw OR 'positron emission tomograph*':ti,ab,kw OR 'positron-emission tomography imaging':ti,ab,kw OR 'pet scan*':ti,ab,kw OR 'pet imaging*':ti,ab,kw OR 'near-infrared spectroscop*':ti,ab,kw OR 'near infrared spectroscop*':ti,ab,kw OR 'nir spectroscop*':ti,ab,kw OR 'near-infrared spectrometr*':ti,ab,kw OR 'near infrared spectrometr*':ti,ab,kw OR 'functional near-	

		infrared spectroscopy':ti,ab,kw OR 'time resolved near infrared spectroscop*':ti,ab,kw	
	37	#35 OR #36	
Brain			
	38	'brain'/exp	
	39	brain:ti,ab,kw OR encephal*:ti,ab,kw OR prosencephalon:ti,ab,kw OR mesencephalon:ti,ab,kw OR rhombencephalon:ti,ab,kw OR cerebrum:ti,ab,kw OR cerebellum:ti,ab,kw OR brainstem:ti,ab,kw OR pons:ti,ab,kw OR cerebra*:ti,ab,kw OR intracranial:ti,ab,kw OR cerebrovascular:ti,ab,kw OR neurovascular:ti,ab,kw OR cranial:ti,ab,kw OR intracerebral:ti,ab,kw OR neuro*:ti,ab,kw	
	40	#38 OR #39	
Imaging techniques combined with brain			
	41	#37 AND #40	
Neuroimaging OR Imaging techniques combined with brain			
	42	#34 OR #41	
Sets combined			
	43	#31 AND #42	
Exclusion of conference abstracts			
	44	#43 NOT 'conference abstract'/it	
Limit to English language			
	45	#43 NOT 'conference abstract'/it AND [english]/lim	1532

Web of Science Core Collection via Clarivate, first run on 2023-06-20, updated on 2024-01-09 and 2025-08-05.

TS= title, abstract and author keywords.

Indexes:

- WOS.IC: 1993 to 2025

- WOS.CCR: 1985 to 2025

- WOS.SCI: 1900 to 2025

- WOS.AHCI: 1975 to 2025

- WOS.BHCI: 2005 to 2025

- WOS.BSCI: 2005 to 2025

- WOS.ESCI: 2005 to 2025

- WOS.ISTP: 1990 to 2025

- WOS.SSCI: 1900 to 2025

- WOS.ISSHP: 1990 to 2025

Search terms			Number of results
Probiotics			
	1	TS=("Escherichia coli Nissle" OR "Escherichia coli strain Nissle")	
	2	TS=("Bifidus")	
	3	TS=("Weizmannia coagulans")	
	4	TS=("Streptococcus salivarius" OR "Streptococcus thermophilus")	
	5	TS=(Saccharomyce* or "CBS 5926" or "baker yeast" or "baker`s yeast" or "brewer yeast" or "brewer`s yeast" or "s cerevisiae" or "saccaromyces cerevisiae")	
	6	TS=("Propionibacterium" or "bacterium acidi propionici" or "Bacillus acnes" or "Corynebacterium acnes" or "Corynebacterium parvum" or "Corynebacterium parvum infectiosum" or "Cutibacterium acnes" or "P. acnes" or "Propionibacteria acnes" or "Propionicibacterium acnes" or "Propionobacterium acnes" or "Proprionibacterium acnes" or "strain CN 6134" or "Bacterium acidi propionici a" or "Bacterium acidi propionici d" or "Propionicibacterium freudenreichii" or "Propionicibacterium freudenreichii shermanii" or "Bacille granuleux" or "Corynebacterium granulosum" or "Propionicibacterium granulosum")	
	7	TS=("Pediococcus pentosaceus")	
	8	TS=("Limosilactobacillus reuteri" or "Lactobacillus fermentum reuteri" or "Limosilactobacillus reuteri" or "Lactobacillus reuteri")	
	9	TS=("Limosilactobacillus fermentum" or "Bacillus casei d" or "Bacillus d" or "fermenti, lactobacillus" or "Lactobacillus	

		cellobiosus" or "lactobacillus fermenti" or "lactobacillus fermentii" or "Lactobacterium fermentum" or "Limosilactobacillus fermentum" or "Lactobacillus fermentum")	
	10	TS=(Lactococcus or "Enterococcus seriolicida" or "Enterococcus serolicida" or "Streptococcus garvieae" or "Bacterium lacti*" or "Streptococcus lactis")	
	11	TS=("bacillus" or "clostridium licheniforme" or "Denitrobacillus licheniformis" or "Bacillus licheniformis" or "b. subtilis" or "Bacillus natto" or "Bacillus subtilis spore" or "Bacillus uniflagellatus" or "Bacterium subtilis" or "Vibrio subtilis" or "Bacillus subtilis" or "Alkalihalobacillus clausii")	
	12	TS=(Lacticaseibacillus or Lactobacillus casei or "Bacillus a" or "Bacillus casei a" or "Bacterium casei a" or "casei, lactobacillus" or "caseobacterium vulgare" or "lactobacilli casei" or "Lactobacterium casei" or "streptobacterium casei" or "Lactobacillus casei")	
	13	TS=(Bifidobacter* or "Actinobacterium bifidum" or "Actinomyces bifidus" or "Actinomyces parabifidus" or "Bacillus bifidus" or "Bacterium bifidum" or "Bacteroides bifidus" or "Bifidibacterium bifidum" or "Cohnistreptothrix bifidus" or "Nocardia bifida" or "Tissieria bifida")	
	14	TS=(Lactobacill* or "Betabacteri*" or "Lactobacileae" or Lactobacter* or acidophilus* or "enpac" or "lacfer" or "lacteol" or "lactophil" or "Thermobacterium intestinale" or "viacil" or "Bacillus casei g" or "Bacillus g" or "Betabacterium breve" or "Levilactobacillus brevis" or "Bacterium curvatum" or "Bacillus acidificans longissimus" or "Bacillus delbrucki" or "bacillus delbrueckii" or "Bacterium delbrucki" or "Plocamobacterium delbrucki" or "Thermobacterium cereale" or "Ulvina delbrucki" or "Bacillus bulgaricus" or "Bacillus lactis acidi" or "Thermobacterium lactis" or "Bacillus casei e" or "Bacillus e" or "Caseobacterium e" or "Plocamobacterium helveticum" or "thermobacterium helveticum" or "Streptobacterium plantarum")	
	15	TS=(probiotic* or psychobiotic* or pro-biotic* or psycho-biotic*)	
	16	#1 OR #2 OR #3 OR #4 OR #5 OR #6 OR #7 OR #8 OR #9 OR #10 OR #11 OR #12 OR #13 OR #14 OR #15	
Neuroimaging			
	17	TS=("neuro imag*" or neuroimag* or "brain activit*" or "brain electrical activity mapping" or "brain cortical thickness" or magnetoencephalography or magneto-encephalography or	

		magnetoencephalogram* or "diffusion tensor imag*" or "diffusion tensor magnetic resonance imaging" or "diffusion tensor mri" or "dti mri" or "diffusion tractograph*" or "functional brain imag*" or "brain mapping" or "brain radiography" or "brain scinitscanning" or neurography or connectome or "dopaminergic mapping" or neuroradiology or "cerebral angiography" or echoencephalography or myelography or pneumoencephalography or eeg or electroencephalogram* or electroencephalography or "brain wave*" or brainwave* or "alpha rhythm" or "beta rhythm" or "delta rhythm" or "gamma rhythm" or "theta rhythm" or "cortical synchronization" or "functional connectivity" or "resting state" or "brain activit*")	
Imaging techniques			
	18	TS=("magnetic resonance imaging" or "echo-planar imaging" or "fluorine-19 magnetic resonance imaging" or "magnetic resonance angiography" or "nmr imaging" or "mr tomograph*" or "nmr tomograph*" or "steady state free precession mri" or zeugmatograph* or "chemical shift imaging*" or "chemical shift imaging" or "magnetic resonance image*" or "magnetization transfer contrast imaging" or "mri scan*" or "proton spin tomograph*" or fmri* or "functional mri*" or "functional magnetic resonance imaging" or "nuclear magnetic resonance imaging" or "diffusion weighted imaging" or "echo planar imaging" or "fluid-attenuated inversion recovery imaging" or "functional magnetic resonance imaging" or "multiparametric magnetic resonance imaging" or "T1 weighted imaging" or "T2 weighted imaging" or "emission tomography" or "computer assisted emission tomography" or "photon emission tomography" or "positron emission tomograph*" or "positron-emission tomography imaging" or "pet scan*" or "pet imaging*" or "near-infrared spectroscop*" or "near infrared spectroscop*" or "nir spectroscop*" or "near-infrared spectrometr*" or "near infrared spectrometr*" or "functional near-infrared spectroscopy" or "time resolved near infrared spectroscop*")	
Brain			
	19	TS=(brain or encephal* or prosencephalon or mesencephalon or rhombencephalon or cerebrum or cerebellum or brainstem or pons or cerebra* or intracranial or cerebrovascular or neurovascular or cranial or intracerebral or neuro*)	
Imaging techniques combined with brain			
	20	#18 AND #19	
Neuroimaging techniques OR Imaging techniques combined with brain			

	21	#17 OR #20	
Sets combined			
	22	#21 AND #16	
Limit to English language			
	23	#21 AND #16 and English (Languages)	298

Supplementary Figure 2. Flow chart of the selection process

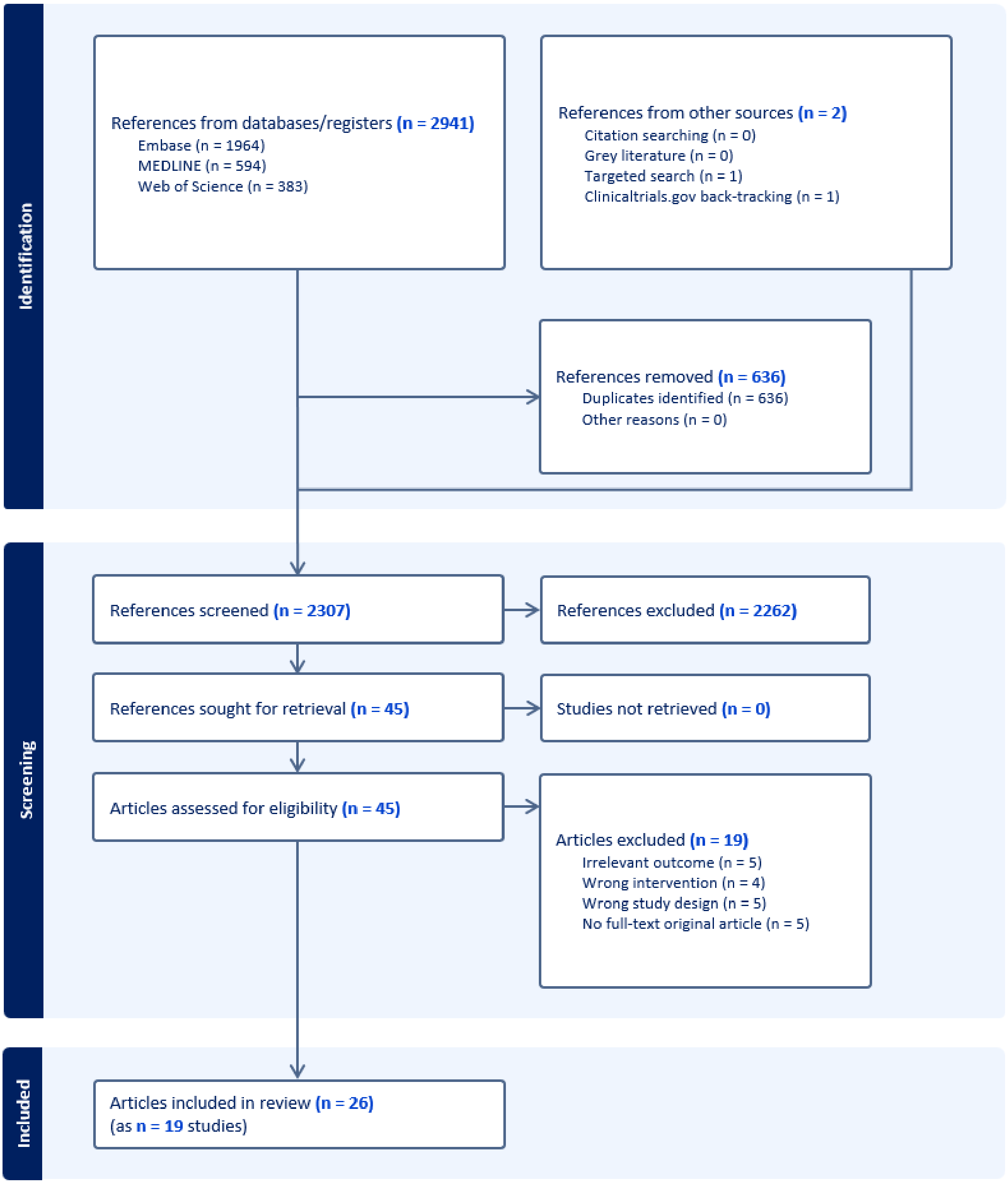

Supplementary Figure

Supplementary Note 3. Risk of bias and study quality assessment – results

The quality of the studies was scored *High* for the majority of the studies for sequence generation[32-33, 35-36, 40-44, 47-57, 81]. In addition, nearly all the studies were scored as *High* and none of the studies was scored as *Low* for blinding of participants and personnel and incomplete outcome data. However, many studies were *Unclear* in reporting descriptions of allocation concealment[34, 37-39, 45-46, 49] and the blinding of outcome assessment[34, 36-38, 45, 47-52, 56-57]. Several of the included studies did not report on all outcomes included in trial registrations or published study protocols[39-40, 48, 52-57, 81] (scored as low quality, if study published before 2023), had little information in trial registration[31-33, 50-51], or did not have a trial registration or study protocol[35-38, 45, 49]. Therefore, these studies were scored as *Low* or *Unclear* for selective reporting. Finally, the majority of the included studies were scored *Low* for other sources of bias, as several had *Unclear* selective reporting and had a sponsor involved, had potential sponsor involvement, or had potential funding bias[31-34, 36-39, 41-57]. Despite the obvious commercial interest, the vast majority of probiotic intervention studies with neuroimaging outcomes seem to be published no matter their outcome. We identified only two studies that were completed more than two years ago without any results being published yet (see Supplementary Note 7 below). Thus, we can conclude that this field has a low prevalence of publishing bias.

Supplementary Figure 4. Study quality assessment – proportions per category.

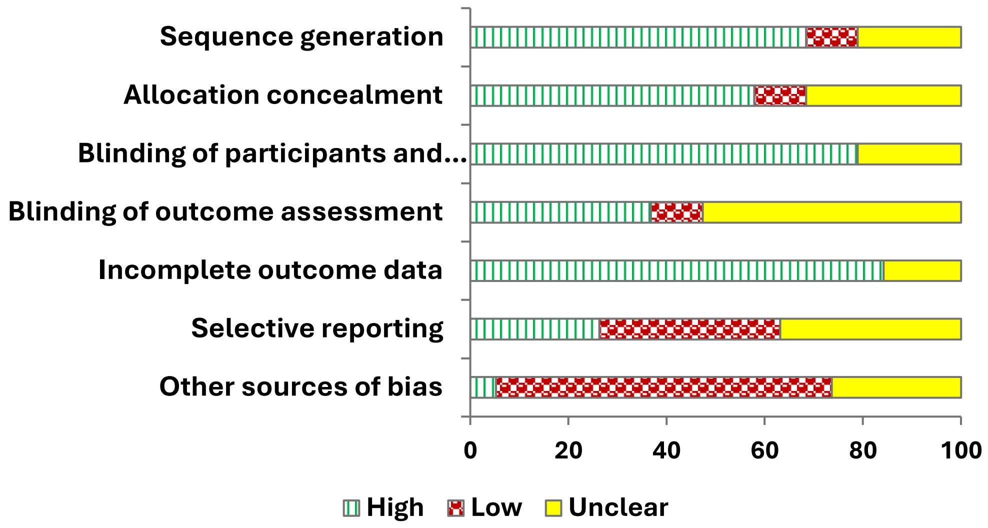

Supplementary Figure. Colours indicate *High* (green) and *Low* (red) quality, and *Unclear* (yellow) indicates that not enough information was available for judgement of study quality.

Supplementary Table 5. Study quality assessment – absolute number of studies per category

High and low indicate quality, and unclear indicate that not enough information was available for judgement of study quality

	**High**	**Low**	**Unclear**
Sequence generation	13	2	4
Allocation concealment	11	2	6
Blinding of participants and personnel	15	0	4
Blinding of outcome assessment	7	2	10

Incomplete outcome data	16	0	3
Selective reporting	5	7	7
Other sources of bias	1	13	5

Supplementary Table 6. Study quality assessment – detailed study-by-study scoring

First author and year of study	Sequence generation	Allocation concealment	Blinding of participants and personnel	Blinding of outcome assessment	Incomplete outcome data	Selective reporting	Other sources of bias
Malaguarnera et al. (2007) [49]	High	Unclear	Unclear	Unclear	High	Low	Unclear
Tillisch et al. (2013) [31]	Low	Low	High	Low	High	Low	Low
Kelly et al. (2017) [34]	Unclear	Unclear	Unclear	Unclear	High	High	Low
Pinto-Sanchez et al. (2017) [50]<br>Martin et al. (2024) [51]	High	High	High	Unclear	High	Unclear	Low
Takada et al. (2017) [36]	High	High	High	Unclear	Unclear	Low	Low
Bagga et al. (2018) [37]<br>Bagga et al. (2019) [38]	Unclear	Unclear	Unclear	Unclear	Unclear	Low	Low
Nakagawa et al. (2018) [45]	Unclear	Unclear	Unclear	Unclear	High	Low	Low
Papalini et al. (2019) [32]<br>Bloemendaal et al. (2021) [33]	High	High	High	High	High	Unclear	Low
Wang et al. (2019) [39]	Low	Unclear	High	High	High	Low	Low
Adikari et al. (2020) [35]	High	Low	High	Low	Unclear	Low	High
Ho et al. (2021) [46]	Unclear	Unclear	High	High	High	High	Unclear

Asaoka et al. (2022) [48]	High	High	High	Unclear	High	Unclear	Low
Ascone et al. (2022) [40, 81]	High	High	High	High	High	Unclear	Unclear
Edebol-Carlman et al. (2022) [41]<br><br>Rode et al. (2022) [42]<br><br>Rode et al. (2022) [43]	High	High	High	High	High	High	Low
Schaub et al. (2022) [53]<br><br>Yamanbaeva et al. (2023) [54]<br><br>Schneider et al. (2023) [55]	High	High	High	High	High	Unclear	Unclear
Kikuchi-Hayakawa et al. (2023) [47]	High	High	High	Unclear	High	High	Low
Mutoh et al. (2023) [44]	High	High	High	High	High	High	Low
Ranisavljev et al. (2025) [52, 57]	High	High	High	Unclear	High	Unclear	Low
Li et al. (2025) [56]	High	High	High	Unclear	High	Unclear	Unclear

Supplementary Note 7. Assessment of potential publication bias – results

On clinicaltirals.gov, we identified a small number of studies conducted in participants aged 18 years and older whose neuroimaging outcomes were not published. Studies assessing populations including participants with a lower age were excluded from the judgement, even if the age range spanned above the age of 18.

One study was found to be completed in 2024 (NCT06013072), back-tracking via the NCT number provided its publication (Ranisavljev 2025[52, 57]). The results of this study have been included in this review, see main text.

One study with status “completed” in 2017 (NCT02417454) as well as one study with status “unknown” and estimated study completion date in 2021 without update (NCT03877393, protocol published as Karakula-Juchnowicz 2019[74]), do not seem to be published. One study (with published protocol Wallace 2020[74]) stated termination due to covid-19 in 2021 (NCT03277586) and another study was withdrawn in 2016 due to unsuccessful recruitment (NCT02552862).

One study with status “completed” in 2020, so far published baseline data but no intervention outcomes (NCT05029765, Cardelo 2022[76]). One study with the status “completed” and an actual study completion date in 2022 (NCT03991195) and one completed in 2024 (NCT05801042) were not found to be published yet. The neuroimaging outcomes of three

studies with status “completed” and an actual study completion date in 2022 (NCT03893162, NCT04823533) or 2023 (NCT05145881) were found to be not published yet, however, the results of other outcome parameters had been published (Nikolova 2023[78], Nikolova 2025[79], Morales-Torres 2023[80], Hsu 2023[77]). Several studies with estimated completion dates in the future seem to be ongoing. We are aware that it takes time from study completion to publication, hence we predict that the majority of those trials will be published soon.

Supplementary Table 8. Additional information on probiotic interventions such as commercial product name, product manufacturer and study funding source

Study	Probiotic strain(s)	Commercial name and manufacturer of the study product	The study products / probiotic strains provided by	Company involvement in the study
Studies analysing single-strain products				
Malaguarnera et al. (2007) [49]	*Bifidobacterium longum* W11	Zirfos, manufactured by Alfa Wasserman, Italy	-	Funding sources not reported.
Kelly et al. (2017) [34]	*Lacticaseibacillus rhamnosus*	-	The study products were provided by Alimentary Health Ltd, Ireland.	One of the authors is affiliated to Alimentary Health Ltd, Ireland
Takada et al. (2017) [36]	*Lacticaseibacillus paracasei* Shirota YIT 9029	-	The probiotic strain was obtained from the Culture Collection Research Laboratory of Yakult Central Institute, Japan.	The study received funding from Yakult Honsha Co., Ltd, Japan.<br><br>Eight of the authors received salaries from Yakult Honsha Co., Ltd, Japan.
Pinto-Sanchez et al. (2017) [50]<br><br>Martin et al. (2024) [51]	*Bifidobacterium longum* NCC3001	-	-	The study received funding from Nestlé SA, Switzerland.<br><br>Two of the authors were employed by Nestec SA, Switzerland.<br><br>Two of the authors were employed by

				Nestlé Institute of Health Sciences SA.
Nakagawa et al. (2018) [45]	*Lactobacillus helveticus* MIKI-020	-	MIKI Corporation, Japan	The study received funding from MIKI Corporation, Japan.<br><br>Three of the authors were employed by MIKI Corporation, Japan.
Wang et al. (2019) [39]	*Bifidobacterium longum* 1714™	1714-Serenitas, manufactured by Alimentary Health Ltd, Ireland	The study products were provided by Alimentary Health Ltd, Ireland.	One of the authors is employed by Alimentary Health Ltd, Ireland.
Adikari et al. (2020) [35]	*Lacticaseibacillus paracasei* Shirota	Commercially available, manufacturer not named	-	No
Ho et al. (2021) [46]	*Lactiplantibacillus plantarum* PS128	-	The study products s were provided by Bened Biomedical Co. Ltd., Taiwan.	No
Asaoka et al. (2022) [48]	*Bifidobacterium breve* MCC1274	-	The probiotic strain was provided by Morinaga Milk Industries Co., Ltd, Japan.	Two of the authors were employed by Morinaga Milk Industries Co., Ltd, Japan.
Kikuchi-Hayakawa et al. (2023) [47]	*Lacticaseibacillus paracasei* Shirota YIT 9029	-	The probiotic strain was obtained from the Culture Collection Research Laboratory of Yakult Central Institute, Japan.	All authors were employed by Yakult Honsha Co., Ltd, Japan.
Mutoh et al. (2023) [44]	*Bifidobacterium breve* M-16V	-	The study products were provided by Morinaga Milk	The study received funding from

			Industries Co., Ltd, Japan.	Morinaga Milk Industries Co., Ltd, Japan.<br><br>Six of the authors were employed by Morinaga Milk Industries Co., Ltd, Japan.
Li et al. (2025) [56]	*Pediococcus acidilactici* CCFM6432	-	The probiotic product was developed by the School of Food Science and Technology and produced by the Yangzhou Institute of Food Biotechnology, both at Jiangnan University, China.	Publicly funded.
Studies analysing multi-strain products				
Tillisch et al. (2013) [31]	*Bifidobacterium lactis*<br><br>*Lactobacillus bulgaricus*<br><br>*Lactococcus lactis*<br><br>*Streptococcus thermophilus*	-	The product was prepared at Danone Research, France.	The study received funding from Danone Research, France.<br><br>Three of the authors were employed by Danone Research, France.
Bagga et al. (2018) [37]<br><br>Bagga et al. (2019) [38]	*Bifidobacterium bifidum* W23<br><br>*Bifidobacterium lactis* W51<br><br>*Bifidobacterium lactis* W52<br><br>*Lacticaseibacillus casei* W56	OmniBiotic® Stress Repair, manufactured by Institut Allergosan, Austria.<br><br>Probiotic mix in the product called Ecologic®825, manufactured by Winclove Probiotics, the Netherlands.	-	Study received funding from Institut Allergosan, Austria and<br><br>Winclove Probiotics, the Netherlands.

	Lacticaseibacillus paracasei W20<br><br>*Lactiplantibacillus plantarum* W62<br><br>*Lactobacillus acidophilus* W22<br><br>*Lactococcus lactis* W19<br><br>*Ligilactobacillus salivarius* W24			
Papalini et al. (2019) [32]<br><br>Bloemendaal et al. (2021) [33]	*Bifidobacterium bifidum* W23<br><br>*Bifidobacterium lactis* W51<br><br>*Bifidobacterium lactis* W52<br><br>*Lacticaseibacillus paracasei* W56<br><br>*Lactobacillus acidophilus* W37<br><br>*Lactococcus lactis* W19<br><br>*Lactococcus lactis* W58<br><br>*Levilactobacillus brevis* W63<br><br>*Ligilactobacillus salivarius* W24	Ecologic®Barrier, manufactured by Winclove Probiotics, the Netherlands.	-	The study received funding from Winclove Probiotics, the Netherlands.<br><br>One of the authors is employed by Winclove Probiotics, the Netherlands.
Ascone et al. (2022) [40, 81]	*Bifidobacterium breve*<br><br>*Bifidobacterium lactis* NCIMB 30435<br><br>*Bifidobacterium lactis* NCIMB 30436	Vivomixx®, manufactured by Mendes S.A., Switzerland	-	No

	Lacticaseibacillus paracasei<br><br>*Lactiplantibacillus plantarum*<br><br>*Lactobacillus acidophilus*<br><br>*Lactobacillus helveticus*<br><br>*Streprococcus thermophilus*			
Edebol-Carlamn et al. (2022) [41]<br><br>Rode et al. (2022) [42]<br><br>Rode et al. (2022) [43]	*Bifidobacterium longum* R0175<br><br>*Lactiplantibacillus plantarum* R1012<br><br>*Lactobacillus helveticus* R0052	Puraflor, GSK Consumer Healthcare, Italy, manufactured by SIIT S.r.l., Italy	-	The study received funding from Pfizer Consumer Healthcare.<br><br>One of the authors was employed by Pfizer Consumer Healthcare.
Schaub et al. (2022) [53]<br><br>Yamanbaeva et al. (2023) [54]<br><br>Schneider et al. (2023) [55]	*Bifidobacterium breve* NCIMB 30441<br><br>*Bifidobacterium lactis* NCIMB 30435<br><br>*Bifidobacterium lactis* NCIMB 30436<br><br>*Lacticaseibacillus paracasei* NCIMB 30439<br><br>*Lactiplantibacillus plantarum* NCIMB 30437<br><br>*Lactobacillus acidophilus* NCIMB 30442<br><br>*Lactobacillus helveticus*	Vivomixx®, manufactured by Mendes S.A., Switzerland	The probiotic product was provided by Mendes S.A., Switzerland.	No

	Streptococcus thermophilus NCIMB 30438			
Ranisavljev et al. (2025) [52,57]	*Lacticaseibacillus rhamnosus* DSM 32550<br><br>*Lactiplantibacillus plantarum* DSM 34532 *(*formerly known as *L. plantarum TIFN101)*<br><br>*Bifidobacterium lactis* DSM 32269<br><br>*Bifidobacterium longum* DSM 32946	Some part of the product is part of *Humiome®,* manufactured by DSM Firmenich AG, Switzerland.	The study products were provided by DSM-Firmenich, Switzerland.	The study received funding from DSM Nutritional Products AG.<br><br>One of the authors was employed by DSM Nutritional Products AG, affiliation DSM-Firmenich, Health, Nutrition & Care.

Supplementary Table 9. Overview of analyses bases applied in the various studies for the assessment of intervention effects on brain health outcomes

	**Whole-brain analysis?**	**Selected brain regions included in targeted analyses**
Studies utilising MRI, fMRI, ASL, DTI		
Tillisch et al. (2013) [31]	yes	Additionally, amygdala, insula subregions, somatosensory regions<br><br>Posthoc analyses with data-driven seed selection
Pinto-Sanchez et al. (2017) [50]<br>Martin et al. (2024) [51]	yes	Additionally, amygdala
Bagga et al. (2018) [37]<br><br>Bagga et al. (2019) [38]	yes	no
Papalini et al. (2019) [32]<br>Bloemendaal et al. (2021) [33]	yes	no
Asaoka et al. (2022) [48]	yes	Additionally, study-specific, selected by data-driven approach
Ascone et al. (2022) [40, 81]	yes	Additionally, hippocampus
Edebol-Carlman et al. (2022) [41]<br><br>Rode et al. (2022) [42]<br><br>Rode et al. (2022) [43]	Yes	Additionally:<br><br>For rsfMRI: default mode network, salience network, frontoparietal network, language network, cerebellar network, dorsal attention network, sensorimotor network, visual network

		For EAT#: precuneus, amygdala, thalamus, (para)hippocampus, caudate, putamen, cingulate gyrus, temporal gyrus, prefrontal cortex, frontal cortex, insula<br><br>MIST#: thalamus, insula, caudate, putamen, cingulate cortex, temporal gyrus, orbital cortex, prefrontal cortex<br><br>#reported whole-brain results, but draw conclusions on regions of interest
Schaub et al. (2022) [53]<br>Yamanbaeva et al. (2023) [54]<br>Schneider et al. (2023) [55]	For emotional task: yes<br><br>For working memory task: yes<br><br>For voxel-based morphometry analyses: yes	For DTI: Uncinate fasciculus tract<br><br>For rsfMRI# and ASL:<br><br>Orbitofrontal cortex, temporal pole, insula, hippocampus, amygdala, subcallosal cortex/subgenual anterior cingulate cortex<br><br>Addtionally, working memory task: hippocampus<br><br>#Posthoc region of interest analyses
Studies utilising fNIRS		
Mutoh et al. (2023) [44]	yes	Focused on reporting prefrontal laterality index
Studies utilising MEG		
Wang et al. (2019) [39]	yes	no
Studies utilising EEG		
Malaguarnera et al. (2007) [49]	yes	no
Kelly et al. (2017) [34]	yes	no
Takada et al. (2017) [36]	yes	no
Nakagawa et al. (2018) [45]	yes	no
Adikari et al. (2020) [35]	yes	no
Ho et al. (2021) [46]	yes	no
Kikuchi-Hayakawa et al. (2023) [47]	yes	no
Li et al. (2025) [56]	yes	no
Studies utilising MRS		
Ranisavljev et al. (2025) [52, 57]	no	Thalamus, frontal, precentral, paracentral, and parietal white and grey matter

MRI: magnetic resonance imaging; fMRI: functional magnetic resonance imaging; ASL: arterial spin labelling; DTI: diffusion tensor imaging; fNIRS: functional near-infrared spectroscopy; MEG: magnetoencephalography; EEG: electroencephalography; MRS: magnetic resonance spectroscopy.

PRISMA 2020 Checklist

Supplementary Table 10. PRISMA checklist

Section and Topic	Item #	Checklist item	Location where item is reported
**TITLE**			
Title	1	Identify the report as a systematic review.	Page 1
**ABSTRACT**			
Abstract	2	See the PRISMA 2020 for Abstracts checklist.	Page 1-2
**INTRODUCTION**			
Rationale	3	Describe the rationale for the review in the context of existing knowledge.	Pages 3-5
Objectives	4	Provide an explicit statement of the objective(s) or question(s) the review addresses.	Pages 3-5
**METHODS**			
Eligibility criteria	5	Specify the inclusion and exclusion criteria for the review and how studies were grouped for the syntheses.	Pages 23-24
Information sources	6	Specify all databases, registers, websites, organisations, reference lists and other sources searched or consulted to identify studies. Specify the date when each source was last searched or consulted.	Page 23
Search strategy	7	Present the full search strategies for all databases, registers and websites, including any filters and limits used.	Pages 23 and Supplementary Note 1
Selection process	8	Specify the methods used to decide whether a study met the inclusion criteria of the review, including how many reviewers screened each record and each report retrieved, whether they worked independently, and if applicable, details of automation tools used in the process.	Pages 23-24
Data collection process	9	Specify the methods used to collect data from reports, including how many reviewers collected data from each report, whether they worked independently, any processes for obtaining or confirming data from study investigators, and if applicable, details of automation tools used in the process.	Page 23-24
Data items	10a	List and define all outcomes for which data were sought. Specify whether all results that were compatible with each outcome domain in each study were sought (e.g. for all measures, time points, analyses), and if not, the methods used to decide which results to collect.	Prospero entry, ID given on page 22<br>Pages 24-25
	10b	List and define all other variables for which data were sought (e.g. participant and intervention characteristics, funding sources). Describe any assumptions made about any missing or unclear information.	Pages 24-25
Study risk of bias assessment	11	Specify the methods used to assess risk of bias in the included studies, including details of the tool(s) used, how many reviewers assessed each study and whether they worked independently, and if applicable, details of automation tools used in the process.	Pages 24-25
Effect measures	12	Specify for each outcome the effect measure(s) (e.g. risk ratio, mean difference) used in the synthesis or presentation of results.	Page 24
Synthesis methods	13a	Describe the processes used to decide which studies were eligible for each synthesis (e.g. tabulating the study intervention characteristics and comparing against the planned groups for each synthesis (item #5)).	Pages 23-24, Table 1
	13b	Describe any methods required to prepare the data for presentation or synthesis, such as handling of missing summary statistics, or data conversions.	-
	13c	Describe any methods used to tabulate or visually display results of individual studies and syntheses.	Page 24
	13d	Describe any methods used to synthesize results and provide a rationale for the choice(s). If meta-analysis was performed, describe the model(s), method(s) to identify the presence and extent of statistical heterogeneity, and software package(s) used.	Page 24

PRISMA 2020 Checklist

Section and Topic	Item #	Checklist item	Location where item is reported
	13e	Describe any methods used to explore possible causes of heterogeneity among study results (e.g. subgroup analysis, meta-regression).	-
	13f	Describe any sensitivity analyses conducted to assess robustness of the synthesized results.	-
Reporting bias assessment	14	Describe any methods used to assess risk of bias due to missing results in a synthesis (arising from reporting biases).	Pages 24-25
Certainty assessment	15	Describe any methods used to assess certainty (or confidence) in the body of evidence for an outcome.	-
**RESULTS**			
Study selection	16a	Describe the results of the search and selection process, from the number of records identified in the search to the number of studies included in the review, ideally using a flow diagram.	Supplementary Figure 2, Page 5
	16b	Cite studies that might appear to meet the inclusion criteria, but which were excluded, and explain why they were excluded.	Pages 17-22
Study characteristics	17	Cite each included study and present its characteristics.	Table 1
Risk of bias in studies	18	Present assessments of risk of bias for each included study.	Supplementary Information 3-6
Results of individual studies	19	For all outcomes, present, for each study: (a) summary statistics for each group (where appropriate) and (b) an effect estimate and its precision (e.g. confidence/credible interval), ideally using structured tables or plots.	Pages 5-16, Tables 1-7
Results of syntheses	20a	For each synthesis, briefly summarise the characteristics and risk of bias among contributing studies.	-
	20b	Present results of all statistical syntheses conducted. If meta-analysis was done, present for each the summary estimate and its precision (e.g. confidence/credible interval) and measures of statistical heterogeneity. If comparing groups, describe the direction of the effect.	-
	20c	Present results of all investigations of possible causes of heterogeneity among study results.	-
	20d	Present results of all sensitivity analyses conducted to assess the robustness of the synthesized results.	-
Reporting biases	21	Present assessments of risk of bias due to missing results (arising from reporting biases) for each synthesis assessed.	Supplementary Note 7
Certainty of evidence	22	Present assessments of certainty (or confidence) in the body of evidence for each outcome assessed.	-
**DISCUSSION**			
Discussion	23a	Provide a general interpretation of the results in the context of other evidence.	Pages 17-22
	23b	Discuss any limitations of the evidence included in the review.	Pages 17-22
	23c	Discuss any limitations of the review processes used.	Pages 17-22
	23d	Discuss implications of the results for practice, policy, and future research.	Pages 17-22
**OTHER INFORMATION**			
Registration and protocol	24a	Provide registration information for the review, including register name and registration number, or state that the review was not registered.	Page 22
	24b	Indicate where the review protocol can be accessed, or state that a protocol was not prepared.	Page 22
	24c	Describe and explain any amendments to information provided at registration or in the protocol.	None
Support	25	Describe sources of financial or non-financial support for the review, and the role of the funders or sponsors in the review.	Page 25

PRISMA 2020 Checklist

Section and Topic	Item #	Checklist item	Location where item is reported
Competing interests	26	Declare any competing interests of review authors.	Page 26
Availability of data, code and other materials	27	Report which of the following are publicly available and where they can be found: template data collection forms; data extracted from included studies; data used for all analyses; analytic code; any other materials used in the review.	Page 25

From: Page MJ, McKenzie JE, Bossuyt PM, Boutron I, Hoffmann TC, Mulrow CD, et al. The PRISMA 2020 statement: an updated guideline for reporting systematic reviews. BMJ 2021;372:n71. doi: 10.1136/bmj.n71

For more information, visit: http://www.prisma-statement.org/
